# Bio-mimetic high-speed target localization with fused frame and event vision for edge application

**DOI:** 10.3389/fnins.2022.1010302

**Published:** 2022-11-25

**Authors:** Ashwin Sanjay Lele, Yan Fang, Aqeel Anwar, Arijit Raychowdhury

**Affiliations:** ^1^School of Electrical and Computer Engineering, Georgia Institute of Technology, Atlanta, GA, United States; ^2^Department of Electrical and Computer Engineering, Kennesaw State University, Marietta, GA, United States

**Keywords:** high-speed target tracking, accuracy-speed tradeoff, ego-motion cancelation, design space exploration, retinomorphic systems, hybrid neural network, event camera, neuromorphic vision

## Abstract

Evolution has honed predatory skills in the natural world where localizing and intercepting fast-moving prey is required. The current generation of robotic systems mimics these biological systems using deep learning. High-speed processing of the camera frames using convolutional neural networks (CNN) (frame pipeline) on such constrained aerial edge-robots gets resource-limited. Adding more compute resources also eventually limits the throughput at the frame rate of the camera as frame-only traditional systems fail to capture the detailed temporal dynamics of the environment. Bio-inspired event cameras and spiking neural networks (SNN) provide an asynchronous sensor-processor pair (event pipeline) capturing the continuous temporal details of the scene for high-speed but lag in terms of accuracy. In this work, we propose a target localization system combining event-camera and SNN-based high-speed target estimation and frame-based camera and CNN-driven reliable object detection by fusing complementary spatio-temporal prowess of event and frame pipelines. One of our main contributions involves the design of an SNN filter that borrows from the neural mechanism for ego-motion cancelation in houseflies. It fuses the vestibular sensors with the vision to cancel the activity corresponding to the predator's self-motion. We also integrate the neuro-inspired multi-pipeline processing with task-optimized multi-neuronal pathway structure in primates and insects. The system is validated to outperform CNN-only processing using prey-predator drone simulations in realistic 3D virtual environments. The system is then demonstrated in a real-world multi-drone set-up with emulated event data. Subsequently, we use recorded actual sensory data from multi-camera and inertial measurement unit (IMU) assembly to show desired working while tolerating the realistic noise in vision and IMU sensors. We analyze the design space to identify optimal parameters for spiking neurons, CNN models, and for checking their effect on the performance metrics of the fused system. Finally, we map the throughput controlling SNN and fusion network on edge-compatible Zynq-7000 FPGA to show a potential 264 outputs per second even at constrained resource availability. This work may open new research directions by coupling multiple sensing and processing modalities inspired by discoveries in neuroscience to break fundamental trade-offs in frame-based computer vision^1^.

## 1. Introduction

**Figure 1:**
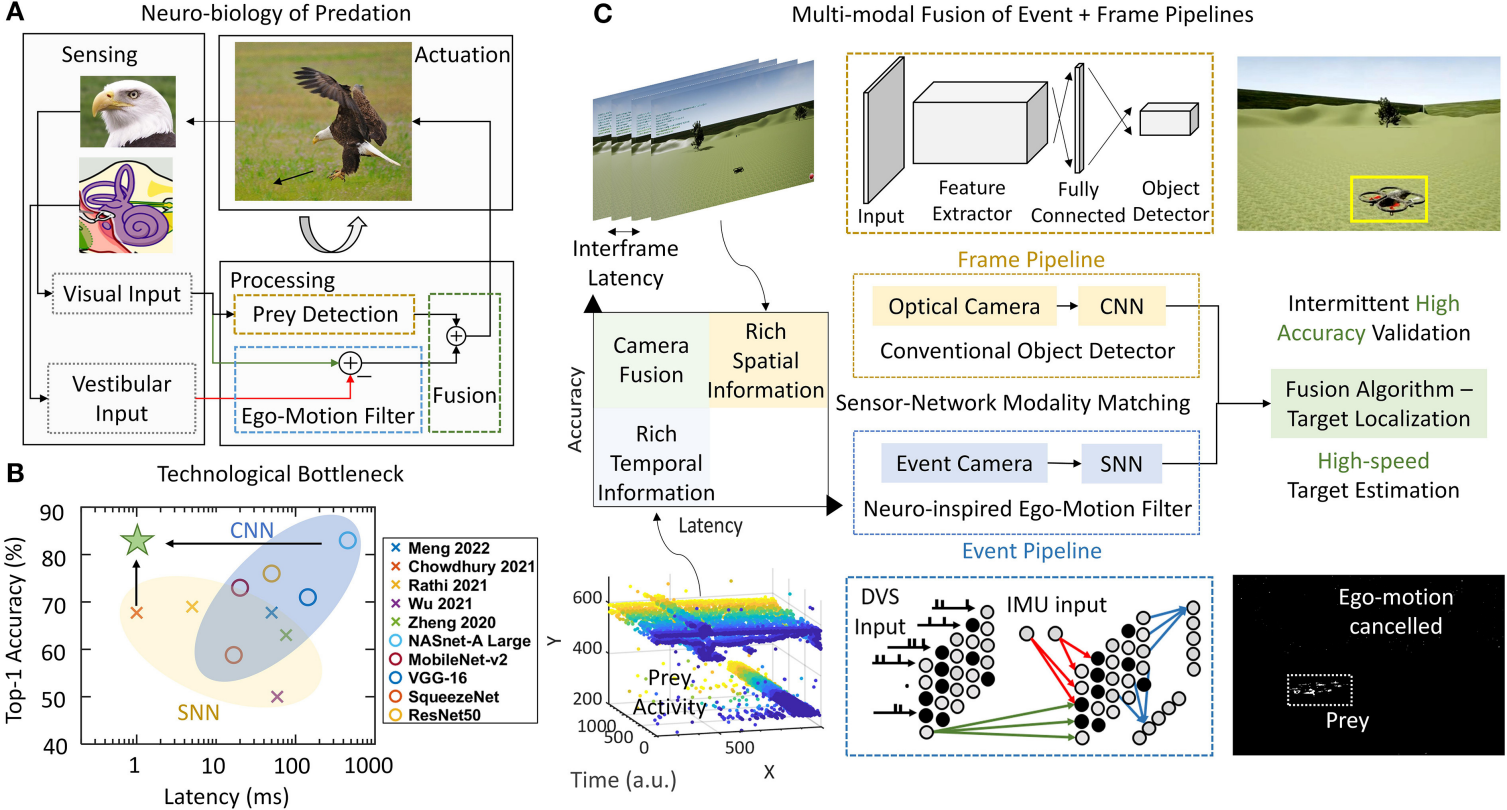
**(A)** Predation combines vision and vestibular inputs to localize the prey in a closed-loop. This involves canceling the self-motion of the predator and identification of the prey. **(B)** Accuracy vs. latency trade-off between SNN and CNN. **(C)** Conventional optical camera + CNN (frame pipeline) for reliable object detection and a parallel event-camera + SNN (event pipeline) for high-speed ego-motion cancelation. The complementary prowess of event and frame processing is fused for target localization. 1. Eagle Eye by TwelveX is licensed under CC BY-NC-SA 2.0. 2. Bald Eagle hunting by vastateparksstaff is licensed under CC BY 2.0. 3. Anatomy of the Human Ear blank.svg by Anatomy_of_the_Human_Ear.svg: Chittka L, Brockmann derivative work: M·Komorniczak -talk- is licensed under CC BY 2.5.

Predatory animals can quickly detect and chase their prey by triggering locomotion to intercept it. Such a behavior involves the visual input for the identification of the prey as well as distinguishing the predator's self-motion (ego-motion) from the relative motion of the steady surroundings (Figure 1A). Cheetahs have been recorded to run at 25 ms^−1^ (Wilson et al., 2013) and their preys also move at comparable speeds within the Field of View (FoV). Successful hunting relies on advanced neural circuits that accept the incoming data from the visual and inertial sensory organs and process it to enable real-time locomotion actuation (Figure 1A). This closed-loop control system across different cortices is capable of highly parallel processing and achieves high power efficiency, speed, and accuracy simultaneously (Sengupta and Stemmler, 2014). Such a biological neural system is optimized over generations through evolution and can be an inspiration to address engineering applications, for instance, the high-speed target localization for autonomous drones under constrained computing resources.

The state-of-the-art method for object detection uses convolutional neural networks (CNN) due to its high accuracy (Zhao et al., 2019; Jiao et al., 2019). Although CNNs are also bio-inspired and have emerged from the layered connectivity observed in primate brain (Cadieu et al., 2014; Khaligh-Razavi and Kriegeskorte, 2014; Güçlü and van Gerven, 2015), the computation gets increasingly intense with larger networks. The models are typically large (Bianco et al., 2018) along with considerable processing latency that puts a limitation on the throughput (outputs per second or frames per second—FPS) of the computation. Light-weight models trade-off the accuracy for latency (Howard et al., 2017). The latency can be reduced while preserving the accuracy by equipping more powerful computing hardware on the drones (Duisterhof et al., 2019; Wyder et al., 2019; Falanga et al., 2020). But the edge computing platforms on a drone usually come with limited accessible power due to the energy density of batteries, which eventually limits the speed and throughput of the computation. Therefore, the traditional frame-based pipeline with frame-based camera (optical camera) and CNNs suffer from the trade-off between computational latency and accuracy for multiple real-time visual tasks including segmentation (Li et al., 2019), object detection (Huang et al., 2017), and gender detection (Greco et al., 2020).

On the other hand, spiking neural networks (SNNs) that represent a new paradigm of artificial neural networks attempt to computationally model biological neural systems. Spiking neural networks exhibit low power consumption in customized hardware platforms (Akopyan et al., 2015; Davies et al., 2018) by the exploitation of asynchronous decentralized tile-based designs. Spiking neural networks have been demonstrated to work for object detection of simple shapes (Cannici et al., 2019) using training methods like approximate backpropagation (Lee et al., 2019; Zhang and Li, 2019) and spike-time-dependent-plasticity (STDP) based training (Diehl and Cook, 2015). Recently proposed bio-mimetic event-based vision cameras called dynamic vision sensors (DVS) boost the potential of SNN-based visual processing even further by matching it with the sensor of a similar modality (Gallego et al., 2019). The regular optical camera lacks in taking full advantage of SNNs because of its discrete frame generation structure where the time-based computation of spikes cannot be fully exploited. The DVS overcome it by allowing continuous-time input generation in the form of events. An event gets generated when the intensity of a pixel in the FoV of the camera changes. Event generation corresponding to all the pixels takes place in parallel and asynchronously, thus sensing only the motion of objects in the FoV saving circuit resources and improving bandwidth. This event-based data flow can be processed by SNNs with matching data modality. Dynamic vision sensor offers low power consumption suited for edge-applications which coupled with high-speed is applied in tasks like robotic goalie (Delbruck and Lang, 2013) and looming object avoidance (Salt et al., 2017). This makes DVS and SNN-based processing (event pipeline) perfectly suited for a task like predation where low-power and high-speed requirements are presented simultaneously.

Spiking neural network frameworks, however, can hardly achieve the same level of detection accuracy compared to their CNN counterparts because of the lack of reliable training methods. Very deep networks cannot be trained easily and reliably because of the non-differentiability of spikes (Lee et al., 2020). Although some attempts using conversion of trained ANN-to-SNN (Kim et al., 2020) provide decent accuracy, the complexity of the network negates the speed advantage of the network. Newer methods with objective functions involving smoothened spikes (Lee et al., 2020) and target spike trains (Shrestha and Orchard, 2018) are proposed but are typically applied to simpler problems (Yin et al., 2021). Spiking neural networks, therefore, lie in the region of low accuracy and low latency (Kim et al., 2020; Cannici et al., 2019). The previous literature shows this clearly as shown in Figure 1B for different SNNs (Chowdhury et al., 2021; Rathi and Roy, 2021; Wu et al., 2021; Zheng et al., 2021; Meng et al., 2022). Simultaneously CNN (Bianco et al., 2018) configurations achieve higher accuracy levels at the cost of slower processing on NVIDIA Jetson TX1. Figure 1B shows results corresponding to Imagenet classification dataset. Imagenet is chosen as it is reasonably complex and is used frequently to benchmark SNN performance. The SNN latency is calculated by using number of time steps for inference with each timestep consisting of 1 ms of synchronization (Merolla et al., 2014). Figure 1B makes it evident that CNNs has a potential to deliver higher accuracy at a lower speed whereas SNNs are capable of providing high-speed if the accuracy can be traded off. Thus, we identify two couples of sensor and processing networks with complementary prowess. Convolutional neural networks and optical cameras show high accuracy by capturing detailed spatial resolution whereas SNN and event-cameras show high-speed processing by capturing of temporal dynamics of the scene. Thus, the processing alternatives present an interesting trade-off between event and frame pipelines (Figure 1B). Our work proposes to overcome this trade-off by using a high-speed ego-motion filter using event pipeline for fast target estimation assisted by the optical camera and CNN based reliable object detection for corroborating the identified position (Figure 1C). The continuously operating SNN filter checks for fast-moving target (prey drone in this case) entering the FoV at all times while the CNN network gets activated at a lower frequency confirming or refuting the presence of the identified prey. The two systems operate in parallel allowing the predator drone to exploit latency and accuracy advantage concurrently. Our approach may find some similarity with the combined event and frame sensing for object detection (Liu et al., 2016) where the event-stream determines the area of interest for CNN. Similarly, another fused approach for optical flow (Lee et al., 2021) combined the sensor outputs in a single CNN-like pipeline. However, both these approaches use CNN backbone. Therefore, the throughput limitation put in by CNN remains and the advantage of event-camera is not fully utilized. Using our multi-pathway approach, we propose to cover a high-resolution spatial domain for prey detection while quickly transferring to a continuous-time domain for high-speed target localization using the insights from the visual systems of predatory animals.

The ego-motion of the moving predator induces events for stationary objects caused by the DVS. Most animals are known to filter out activity caused by ego-motion using different kinds of sensory feedback systems (Kim et al., 2015). It was proposed that vestibular (inertial) feedback signals through inhibitory connections compensate for the ego-motion in insects and primates (Zhang and Bodznick, 2008; Benazet et al., 2016). This was experimentally demonstrated recently, in Kim et al. (2015), where the vestibular sensor induced self-motion cancelation was observed by probing the neurons in houseflies. (1) Our first contribution lies in the design and implementation of a bio-inspired SNN-based ego-motion cancelation filter fusing event-based vision with vestibular and depth information. Our SNN filter removes the activity generated by the ego-motion leaving only the events corresponding to the moving prey by mimicking the neuro-biological counterparts. The loss of accuracy in the noisy SNN filter is compensated by a highly accurate CNN-driven object detector which captures and processes the RGB image periodically to validate the SNN estimate. Therein lies the second key contribution of this article. (2) We propose a close interplay between CNNs and SNNs by coupling spatio-temporal consistency criterion with a neuro-inspired model. This co-ordination between multiple pipelines in different phases of chasing is inspired by the use of specialized neuronal clusters in different phases of hunting in Larvae zebrafish (Förster et al., 2020). The separation between a locally fast (event pipeline) and globally slow signal (frame pipeline) is similar to primate vision (Mazade et al., 2019). Our algorithm relies on a CNN to detect and identify the prey when it is far and a longer detection latency is acceptable, and gradually hands over the task to the SNN as the predator starts to approach the prey and a shorter latency for fast-tracking is of the essence. Our multi-pipeline processing with color information (frame pipeline) for accuracy and motion information (event pipeline) for speed emerges from the similar color and motion separation in visual processing of primate and insect vision (Gegenfurtner and Hawken, 1996; Yamaguchi et al., 2008).

The algorithm is verified in a three-step process. In the first step, we implement it on a programmable drone environment—Programmable Engine for Drone Reinforcement Learning Applications (PEDRA) (Anwar and Raychowdhury, 2020) with different environments of varying level of obfuscation and multiple evasive trajectories for the prey. In the second stage, the algorithm is implemented on a real drone in both indoor and outdoor environments. The prey drone is manually flown in front of the closed-loop autonomous predator drone while the DVS data is emulated from the frame-based images captured by the onboard camera of the predator. Finally, we record a prey flight using a hybrid camera assembly with on-board inertial measurement unit (IMU) and process it to show accuracy preserving high-speed computation tolerating real-world sensor noise. The design space is explored to tune optimal parameters for the SNN, appropriate model for the CNN and their impact on the interplay on the fused CNN+SNN system. Finally, we estimate the circuit level cost of implementing such a system on a edge-compatible FPGA to show a potential throughput of >264 outputs per second. This work shows the conjunction of SNN with more established CNNs for specialized high-speed processing. This work may open a new research direction by coupling parallel sensing and processing modalities to break fundamental trade-offs in frame-based computer vision.

## 2. Methodology

### 2.1. Target estimation - ego-motion cancelation using SNN

Identification of the prey from the cluttered event stream requires separation of events corresponding to ego-motion and their efficient cancelation. Model-based optimization methods like contrast maximization (Gallego and Scaramuzza, 2017; Rebecq et al., 2017), feature tracking (Kueng et al., 2016; Zihao Zhu et al., 2017), or deep learning techniques (Alonso and Murillo, 2019; Mitrokhin et al., 2019) have been used for ego-motion cancelation and moving object detection in event cameras. However, these methods require iterative optimizations and multiple memory accesses lowering the speed of computation. Secondly, our method uses CNN for accuracy compensation. Therefore, high-speed requirement takes precedence over accuracy for event pipeline and we rely on bio-inspired faster alternatives while allowing compromise in accuracy. The performance of a object detection is typically measured using the overlap between the ground truth and predicted bounding boxes. The target localization task at hand requires actuating the predator with appropriate velocity and rotation depending upon the region in which the target is present. Therefore, an accurate detection is the one where the output of SNN and CNN lies within a threshold of pixel distance from the actual position of the prey drone. This easier definition of accuracy allows measurement in terms of percentage of correct localizations as used in the rest of this article.

**Figure 2:**
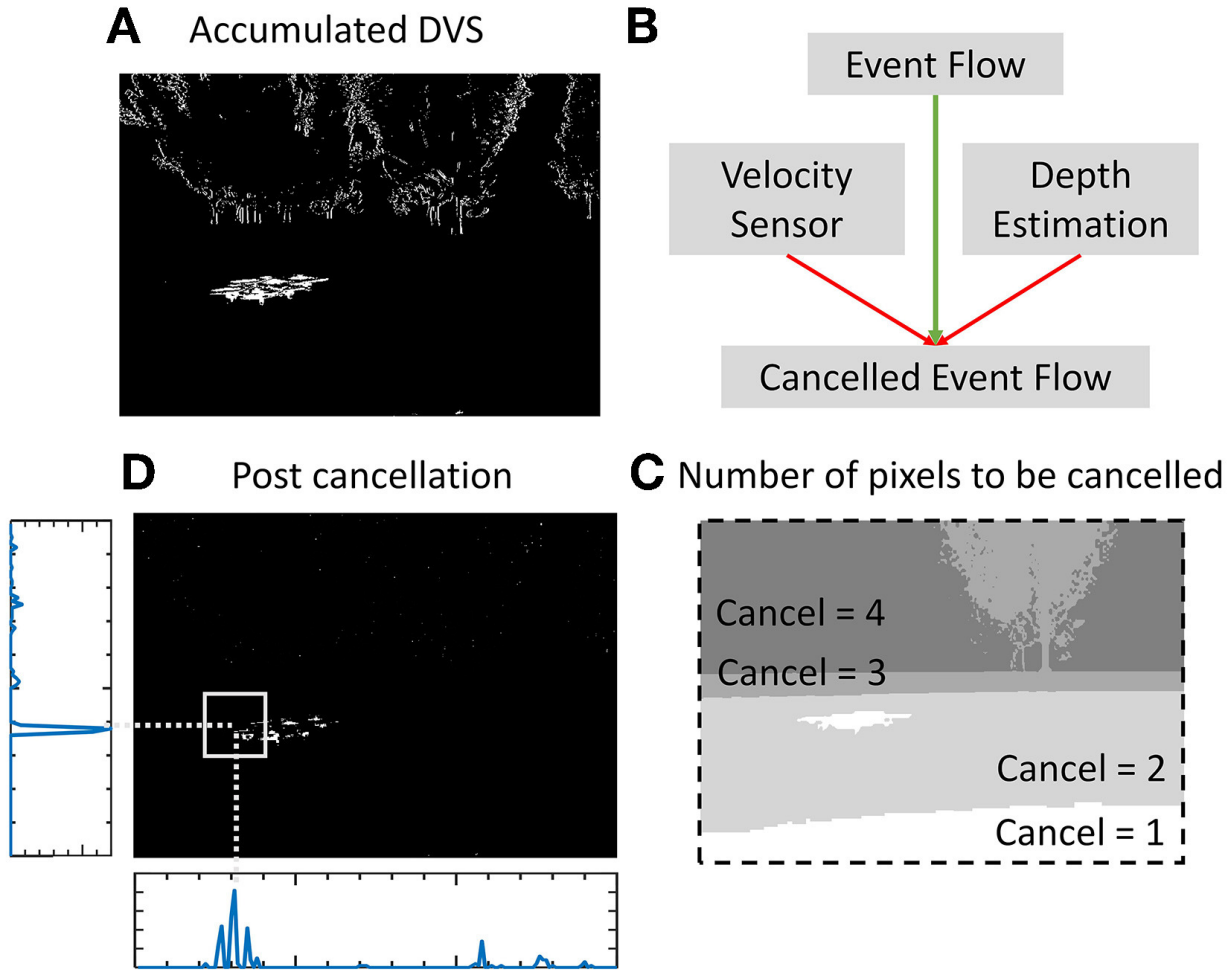
**(A)** Accumulated events within a time window from the event camera. **(B)** High self-velocity and higher depth requires more activity cancelation to preserve the activity of the moving target in close vicinity. **(C)** Number of pixels to be canceled at every position in the image. **(D)** Ego-motion cancelation removing the activity corresponding to the stationary objects with the surviving activity corresponding to the target (prey drone).

The events-accumulated frame generated by the event stream from the event camera in a time window is shown in Figure 2A. The independent rapid motion of the prey creates a denser cluster of events around it as seen in the image. Other events are generated by the stationary objects within the scene and should be canceled. The higher self-velocity of the predator generates more events corresponding to stationary objects. Therefore, activity cancelation needs to be proportional to the predator's self-velocity. Secondly, the reliance on the event pipeline is higher when the prey is close to the predator where it can quickly evade and escape the FoV. This is because the time of escaping the FoV is long when the prey is at a longer distance, and slower detections from frame pipeline are more reliable. Therefore, the SNN filter needs higher accuracy when the distance between the prey and predator is small. Therefore, the events at a higher depth from the predator are canceled out to boost activity in the close vicinity.

This cancelation strategy is illustrated in Algorithm 1. Every continuous patch of active pixels requires a fixed number of events to be canceled from it. This cancel mask is denoted by “cancel.” The pixel array is denoted by “p” where pixel values are either 0 or 1. This is proportional to the self-velocity of the predator and the depth of the pixel undergoing the cancelation operation (Figure 2B). *v*_*H*_ and *v*_*V*_ denote the scalar horizontal and vertical component of the predator motion including velocity and rotation which is called self-velocity in the article. This is acquired through the onboard IMU of the event camera. The depth is acquired from a stereo camera which provides depth for every pixels in meters. The velocity and depth are both normalized using empirically found multipliers to make them dimensionless for addition in Algorithm 1. Figure 2 shows the cancelation strategy with the number of events to be canceled at every position shown in Figure 2C. With the prey motion being faster than the steady environment, the activity corresponding to the prey persists even after the cancelation while the activity corresponding to the stationary background gets canceled. Figure 2D shows the image after canceling out the ego-motion generated events. Horizontal and vertical binned histogram computation of the number of surviving pixels in this image gives the approximate position of the prey.

**Algorithm 1:**
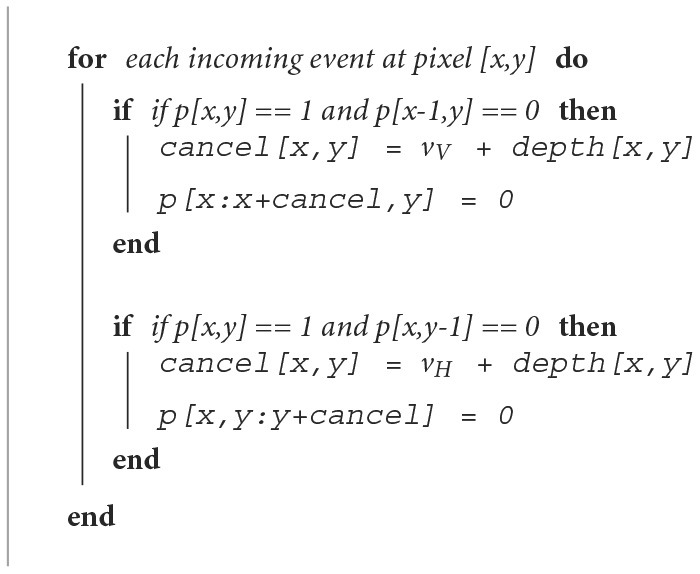
Frame-based self-motion cancelation.

However, this analysis relies on an event accumulated frame-based computation which adds an additional overhead of frame accumulation on the asynchronous event stream from the DVS camera. Processing of the incoming events in the matched asynchronous modality offers higher speed and energy efficiency in the sparse computation effort. This is because accumulating the frame followed by cancelation (matrix operations on *n*×*n* matrix) adds *O*(*n*^2^+*m*) complexity where *m* is the number of events. On the other hand, processing the events independently allows the speed of *O*(*m*). Therefore, we propose a four-layered SNN for processing of Algorithm 1 in real-time. The network gets its inspiration from recent neuro-biological discoveries explained in Section 4.

**Figure 3:**
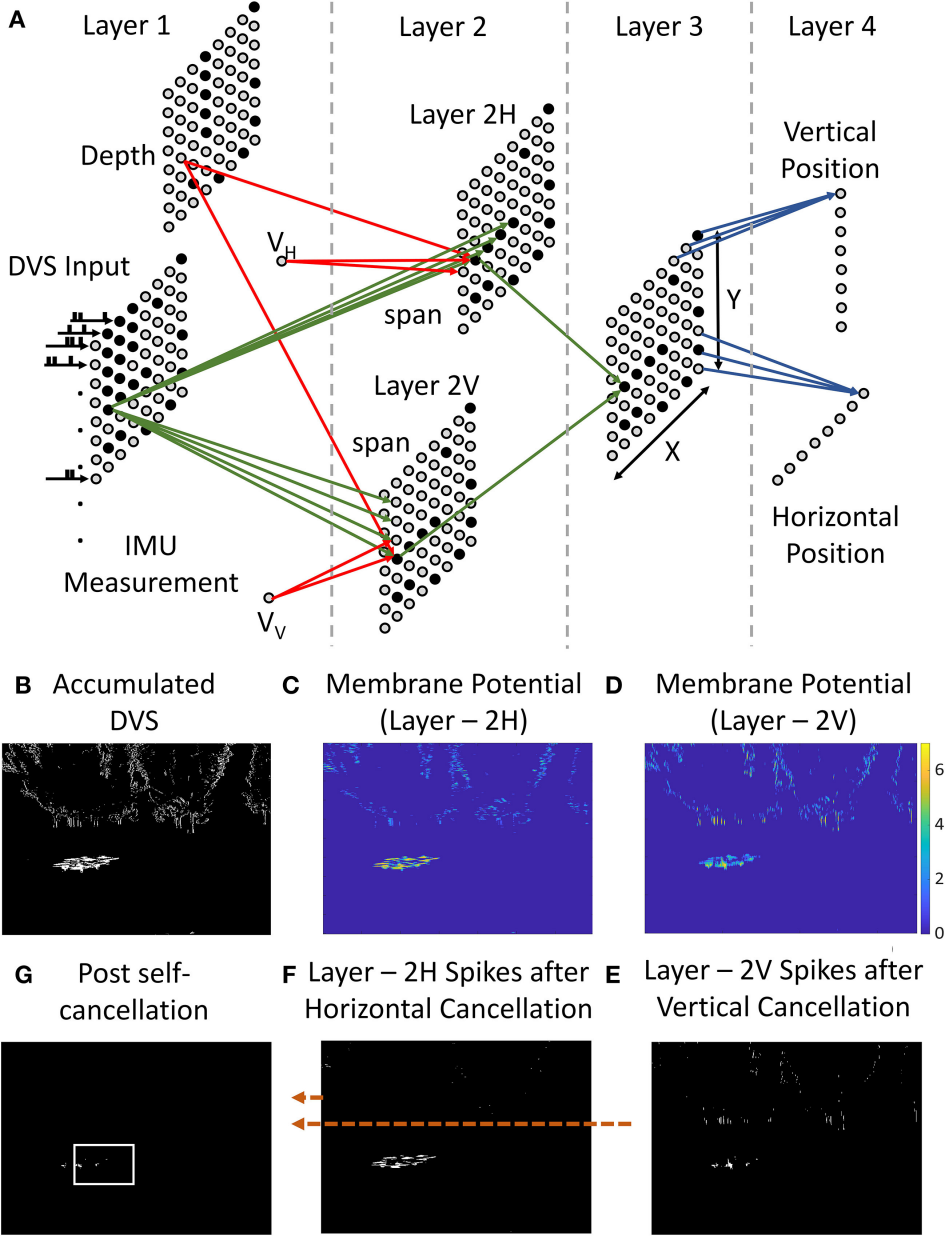
**(A)** Four-layer ego-motion filtering SNN. Event-data, self-velocity, and depth information constitute the input and identified position of the prey is provided at the output. **(B)** Event-accumulated frame within a time window. **(C,D)** Membrane potential of the neurons in layers 2H and 2V. Patches of continuous event activity cause higher membrane potential build-up. This makes patches of high activity likely to spike more. **(E,F)** Spikes issued by the 2H and 2V neurons. The prey activity preferentially survives because of the presence of continuous event patches near the prey. **(G)** Spiking of Layer 3 neurons with AND operation on layer 2V and 2H for SNN output generation.

Every incoming event carries its location (*x*, *y*), time of generation (*t*), and polarity (*p*) feeding to the input layer of the network shown in Figure 3A. Each spiking neuron obeys the integrate and fire (IF) dynamics shown in the following equations.


(1) $$\begin{array}{l} V{\left[x,y\right]}_{t+1}=V{\left[x,y\right]}_{t}+\underset{i}{\sum }W_{i}\left[x,y\right]S_{i}{\left[x,y\right]}_{t} \end{array}$$

(2) $$\begin{array}{l} if\;V{\left[x,y\right]}_{t+1}>V_{th}\;\text{then}\;S{\left[x,y\right]}_{t+1}=1,V{\left[x,y\right]}_{t+2}=0 \end{array}$$

The summation term corresponds to the incoming current from the connected neurons (denoted by *i*) that spiked the previous time instance. The synaptic weight from neuron “*i*” to the neuron being updated ([*x*,*y*]) is denoted by *W*_*i*_ The spiking of a neuron is denoted by *S* where *S* = 1 if the membrane potential exceeds the spiking threshold (*V*_*th*_). The input from the previous synapses drives the output neuron at the immediate next time step. This avoids the incorporation of the synaptic delays and computation of time-delayed currents simplifying the computation.

The first layer takes in the event stream from the event camera (Figure 3A). This is connected to the next layers for vertical (Layer 2V) and horizontal (Layer 2H) event cancelation. Every neuron in the DVS layer drives “span” neurons above it in Layer 2V and “span” neurons on the right of it in Layer 2H with synapses of unit weight. Layer 2 is also driven by velocity encoding neurons and depth encoding neurons. Both velocity neurons and depth neurons are connected using inhibitory synapses. The predator's self-velocity needs to be calculated using accelerometer readings from the IMU in the current step and is converted to multi-neuron spiking activity by discretizing it given by *v*_*H*_ and *v*_*V*_ and is connected to layer-2 using inhibitory synapses. Every velocity neurons is connected to all neurons in layer 2. Depth neurons are connected to the neurons in the same position in layer 2.

For every incoming spike at position [*x*, *y*], the membrane potential for the neurons in layer 2 rises by a fixed amount given by the synaptic weights from the DVS layer while it is pulled down by velocity neuron and depth neurons. Only when a continuous spatial region has persistent activity (Figures 3C,D), the potential rise is enough to cause a spike (Figures 3E,F). This naturally cancels out the noisy cluttered events. The self-velocity and depth for every pixel determine a minimum width of the spatial continuous spiking patch required to trigger spiking in layer-2. Large self-velocity causes more spikes in a patch that need to be removed. Therefore, higher self-velocity requires wider patches of continuous activity to cause spiking in layer 2 and vice versa. The synaptic weights have a unit value for all the excitatory synapses. The negative (inhibitory) weights of the velocity and depth neurons depend critically upon the resolution of the event camera and FoV. They are empirically calculated to ensure exact cancelation of ego-motion when there is no prey drone in the environment. Figure 3 shows the network along with the activation and spiking in each layer. The membrane potentials of the neurons are shown in Figure 3B. Stationary objects have sparser events as shown in Figure 3B causing a small potential rise in layer 2. This causes spiking to be sparse in these regions. Thus, a persistent spiking in layer-2 happens in the region corresponding to the prey drone. Thus, layer-2 carries out the filtering activity as denoted in Algorithm 1 in an asynchronous spiking manner.

The intersection of surviving activity in both layer 2H and layer 2V corresponds to the region of the prey. Layer-3 carries out an AND operation using excitatory connections making the activity survive only when both the layer have spiked in that region. This ensures that only the pixels surviving after the cancelation of both vertical and horizontal motion survive to contribute to the identification of the prey. This is shown in Figure 3G. Layer 4 calculates the pixel with the highest spiking activity by calculating the histograms shown in Figure 2D. All neurons in a row for layer 3 are connected to vertical position neurons in layer 4 and similar connections are used for the horizontal position. High sustained activity within a column/row drives the horizontal/vertical position neuron to spike. The intersection of the maximum spiking activity detected by vertical and horizontal position neurons is declared as the estimated position of the target (prey drone).

Asynchronous incoming events in layer- 1 requires continuous operation of layer-1. However, the actual position of the target need not be updated every microsecond because of the finite mechanical delay in actuating the predator drone. Thus, the layer-3 and layer-4 that infer the presence of the target from the spiking pattern in layer 2 are calculated at a fixed time interval called an epoch which determines the throughput (outputs per second) of the system. The throughput is also called FPS at some points because of its resemblance with the throughput of frame pipeline. At the end of every epoch, layers 2 and 3 are reset back to resting potential. This avoids unnecessary build up of potential from the previous activity from interfering in the future detection in absence of leakiness. It also saves the storage and computation of previous spiking time-stamp for every pixel to calculate the leakage within the neuron for every incoming event. As there is no restriction on frame rate for the DVS, the epoch can be made arbitrarily small increasing the throughput. However, a very small epoch causes a small number of incoming events to infer from with noise leakage causing an accuracy drop. However, the epoch duration is still significantly smaller than the inter-frame time interval of the optical camera giving higher FPS for the SNN pipeline. The trade-off is explored in detail in Section 3.3. All neurons are restored to the reset potential of “0” after an epoch is over. The SNN proves useful when the prey generates a large number of events compared to the background. This condition naturally exists when the prey is close. The accuracy of the SNN degrades gradually as the prey moves farther. However, for prey at a distance, CNN works reliably as the prey cannot escape the FoV quickly and can be tracked.

### 2.2. Prey detection via CNNs

Convolutional neural networks is required to add fault tolerance to the reasonably accurate and fast SNN. Drone detection using CNNs is well-explored (Chen et al., 2017; Nalamati et al., 2019) with different models and training methods having different accuracy vs. latency characteristics (Aker and Kalkan, 2017; Sun et al., 2020; Singha and Aydin, 2021). The CNN provides a bounding box around the drone. The mid-point of the bounding box is used as the CNN output. This provides an anchor position for the fusion algorithm to determine whether the SNN outputs are usable. However, it is important to note that the final task at hand is target localization for closed-loop chasing application. Therefore, the exact dimensions of the bounding box do not have a stringent restriction as required in the previous works where an accurate object detection task is intended. Additionally, the CNN output provides a reasonable estimate of the region of presence of the target within the FoV for actuating the predator platform. The Euclidean distance between the SNN and CNN outputs from the true mid-point of the target's position is used for calculating the accuracy. We fuse the output of the neuroscience-inspired SNN filter with an established electronic CNN pipeline for boosting the throughput of target localization to track evasive target prey. The accuracy vs. latency trade-off within the CNN caused by different models and detection algorithms affects the final accuracy after fusion. Thus, selection of feature detection backbone and detection method forms a key decision. These trade-offs are explored in the section 3.3 and the choice of network is explained.

Reconstruction of intensity image from the events produced by the DVS followed by conventional CNN based-object detection is possible saving additionally required optical camera in our work (Rebecq et al., 2019). Low-cost reconstruction approaches have been demonstrated in Liu and Delbruck (2022) for optical flow calculation where the binary intensity frame is generated by event accumulation followed by block matching for calculating the local optical flow. Mohan et al. (2022) uses event accumulated binary frames for traffic monitoring for detecting moving cars by a stationary event camera. However, our work requires a frame-based accurate target detection using CNN for maintaining the overall accuracy of the system. Thus, we expect that this application will benefit from reliable intensity information requiring accurate event-to-frame reconstruction. These approaches are typically computationally heavy (Wang et al., 2019), consuming vital circuit resources. We, therefore, take the approach with separate optical and event-based cameras in this work.

### 2.3. Target localization - fusing the SNN and CNN outputs

The complementary specialization of event and frame pipelines in capturing the temporal and spatial details make their expertise in accuracy and latency complement each other. The fused system uses either the most recent SNN output or CNN output as the final localized position of the target and uses it to actuate the predator drone for chasing.

When the target has not been “seen” by the CNN, the SNN looks for a suspicious activity with its high speed. The fusion algorithm uses the SNN output as the final localized position of the target if multiple SNN outputs are spatio-temporally consistent with each other. This causes the predator to start chasing the prey drone at the final fused position even before the CNN checks if it is the required target. Thus, the fusion algorithm needs to signal the CNN to confirm whether the activity corresponds to the required target—adding object selectivity for a target. The chasing with SNN detected activity makes sure that the prey does not enter and evade the FoV of the predator before CNN could process it.

Secondly, when the target is in the close vicinity and generates significant activity, the SNN needs to utilize the high-speed output for actuation while the CNN output confirms the prey position it sporadically. When a CNN output is available, the SNN outputs after it use it as an anchor to check their spatio-temporal consistency. Therefore, both SNN and CNN outputs are required to ensure correct chasing—both before and after the presence of the target is confirmed within the FoV. However, one of them is better suited depending upon the distance between the prey and predator as the predator passes through different stages of capturing the prey. These are listed below.

- Case-1 (Finding the prey): The predator rotates around itself to find the prey in the environment around it. Any spurious event activity causes consistent SNN outputs to build suspicion. The CNN also keeps detecting in parallel. If multiple SNN outputs infer the same region (spatio-temporal consistency), then the suspicion level rises beyond a threshold. This indicates the possibility of the prey being present and the predator starts approaching while the CNN is triggered to provide its inference for validation.
- Case-2 (Approaching the prey): A relatively long distance between the predator and prey causes the prey to generate a small number of events in the event camera output. Thus, it is highly likely that this activity gets canceled by the SNN filter. However, the CNN is reliable in this domain because the prey stays in the FoV for a longer time and CNN latency is permissible. This allows the CNN inference to track accurately with a relaxed constraint on latency.
- Case-3 (In the close vicinity of the prey): As the predator approaches the prey, the event activity of the prey increases making the SNN more reliable. Simultaneously, the latency constraint gets stringent as the prey can evade quickly. Therefore, the fusion mechanism works best in this phase. The noisy SNN inference is compared with the CNN inference for spatial continuity and SNN output from the previous epoch for temporal continuity. A spatio-temporal consistent SNN output is declared as the position of the target.

The error compensating fusion scheme is outlined in Algorithm 2. The predator starts by searching for the prey by rotating around itself till the prey is found by either SNN or CNN. At every epoch of processing, the RGB frame and IMU data is captured while the event stream continuously comes in. The SNN filter operates continuously to identify if the prey enters the frame and generated an output after every epoch. Once the activity is detected, the output has to go through a spatio-temporal consistency check with the recent SNN and CNN outputs. This is carried out by defining a suspicion level. If the position identified by the SNN [*position*_*SNN*_(*t*)] at time step “*t*” is close to the most recent CNN detection, then this indicates the spatial continuity with the reliable CNN output and this SNN output is declared as the final fused position (*position*_*fused*_). However, it might be possible that the CNN has not detected the prey yet (*found*_*CNN*_ = 0). In this case, *position*_*SNN*_(*t*) is also compared with the identification of the SNN at the previous epoch *position*_*SNN*_(*t*−1) to check temporal continuity. If the SNN outputs are spatially close within the FoV, the suspicion level rises. This makes sure that the SNN outputs correspond to a genuine external motion in the region. For the suspicion level beyond a threshold, the SNN output is declared as the final fused output.

**Algorithm 2:**
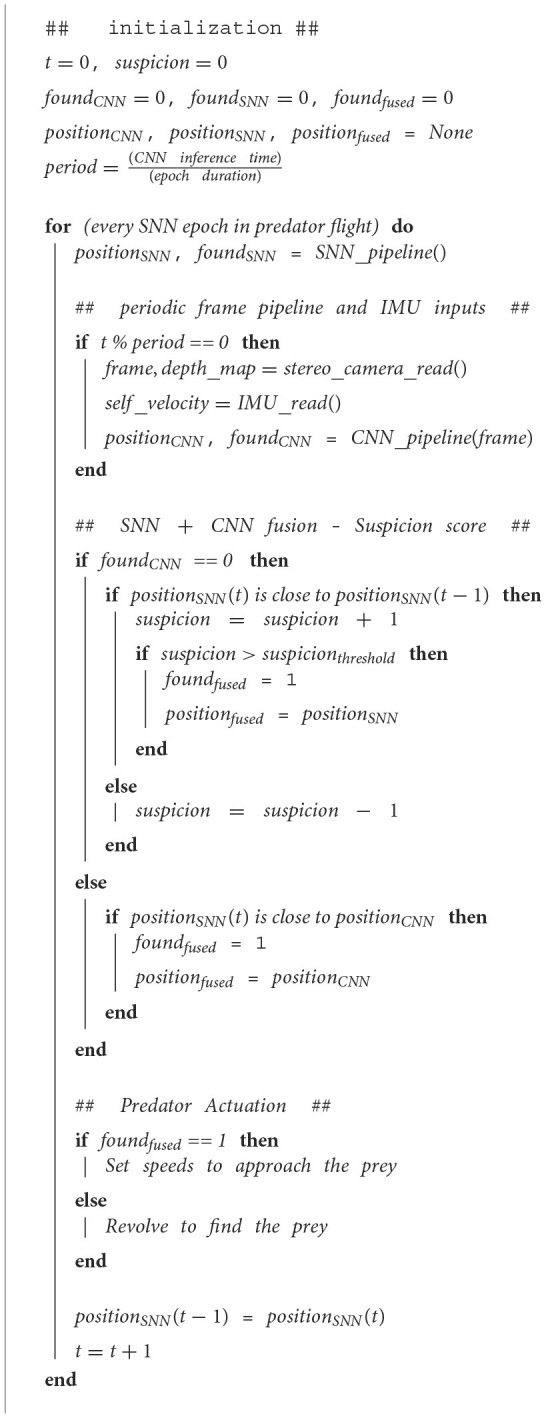
Fusion algorithm.

If the suspicion score rises above the predefined threshold, this also triggers the CNN to confirm that the detection corresponds to the prey. The CNN is also activated after every fixed period of time. The area of the bounding box detected by the CNN is used to estimate the distance between the predator and prey. A larger bounding box corresponds to the prey being in close vicinity. Depending upon the distance between the prey and predator, the relative importance of SNN and CNN are determined. If the prey is close, then most compute resources can be allocated to SNN with sparser CNN validations. Whereas if the prey is far, the CNN is made to operate at maximum throughput by taking compute resources from SNN as required in case-2. Depending upon the position of the prey identified in the FoV, the actuation velocities are selected with the goal of keeping the prey at the center of the frame.

The allocation of computing resources to SNN and CNN by tuning the operating frequency of the CNN dynamically depending upon the distance between the prey and predator assumes the same computing platform being used for the implementation of both SNN and CNN. If the same platform has enough resources to share (e.g., FPGA) for running both pipelines in parallel, then both SNN and CNN can be operated at its maximum throughput and multiple epochs of SNN outputs would be compared with the most recent CNN output for spatial continuity.

## 3. Results

### 3.1. Verification using virtual environments

The autonomous flights of drones within virtual environments are enabled by PEDRA (Anwar and Raychowdhury, 2020). Programmable Engine for Drone Reinforcement Learning Applications connects virtual environments created in Unreal Engine to airsim (Shah et al., 2018) enabled drones through a module-wise programmable python interface. User-defined environments can be created within Unreal Engine with varied level of complexities as used in typical gaming platforms. Multiple drones can be instantiated with a set of image, depth, and inertial sensors mounted on them using airsim. The drones can be actuated at specific velocities and orientations to interact with the environment. The actuation can be pre-programmed for every time step or can be determined by the CNN inference on the images captured by onboard camera. Images can be captured from the point of view of the drone and processed using Tensorflow for image processing for actuating the drone for the next time step. Programmable Engine for Drone Reinforcement Learning Applications provides a training and evaluation framework for the tasks that otherwise cannot be directly tuned on a flying platforms. We instantiate a prey and a predator drone in multiple virtual environments created for this study. As PEDRA only provides frame-based image sensing, we add experimentally calibrated frame to event conversion using v2e tool (Hu et al., 2021). This provides a time-stamp encoded event stream by fine-grained interpolation images and calculation of intensity differences calibrated with real DVS cameras. Thus, both event-based and frame-based visual data is added to existing PEDRA infrastructure. The images and event-stream captured by the predator drone are handed over to the Python backend implementing both SNN and CNN. We program the trajectory of the prey drone while the predator is controlled using the output of the vision backend. We use Intel i9 Processor and NVIDIA Quadro RTX 4000 GPU for the simulation experiments. Both networks provide their outputs as the center point of the detected target that are used in the fusion algorithm to determine the final fused target position.

#### 3.1.1. Operation of fusion algorithm

**Figure 4:**
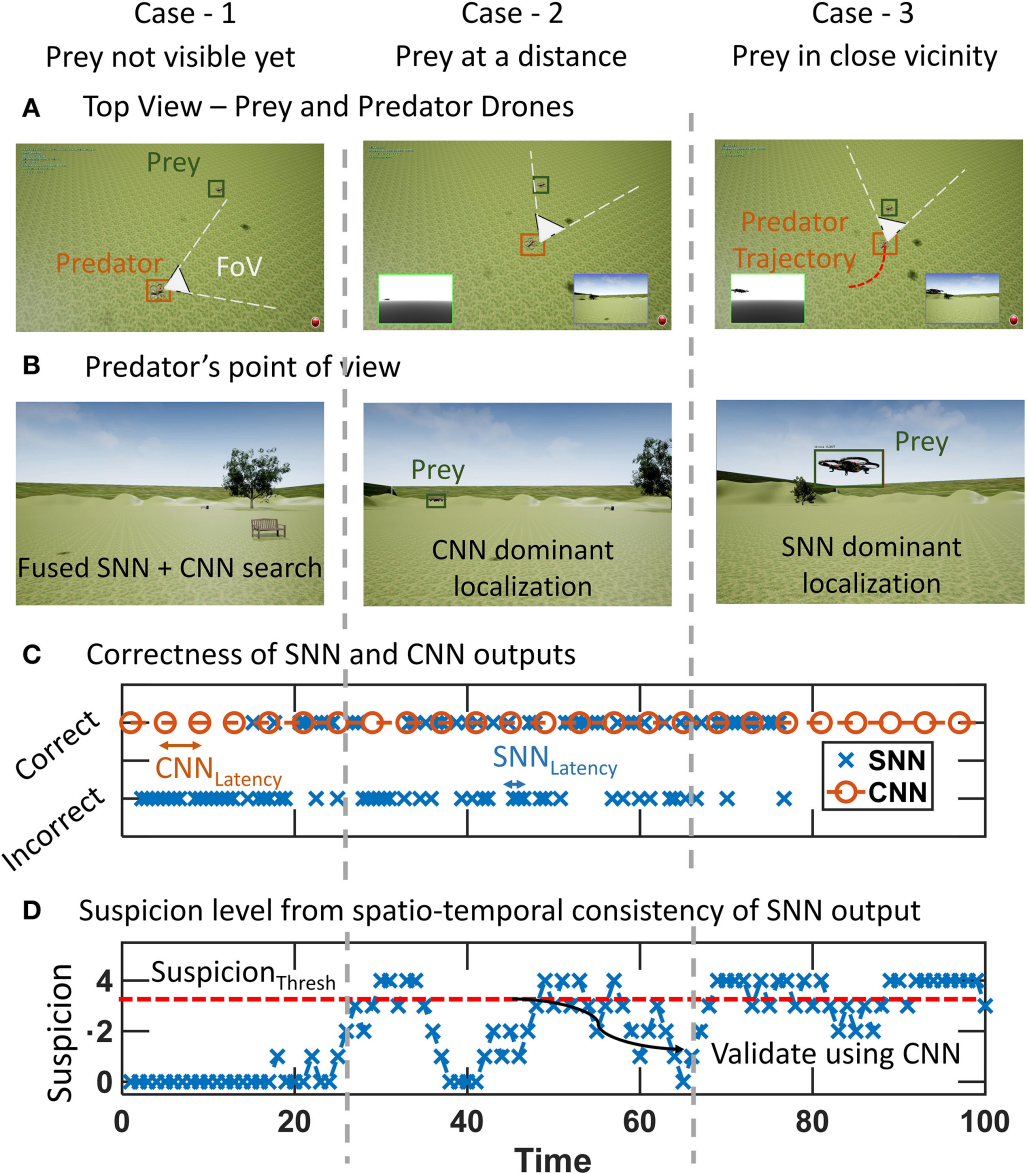
Phases of chasing the prey drone as the predator passes through cases 1–3. A time step corresponds to one output of SNN and is denoted by “time” in figure. **(A)** Top view of the prey and predator drone positions. The prey becomes visible and is approached from case 1 to case 3. **(B)** Predator drone's point of view. **(C)** Correctness of output of SNN and CNN. SNN is more reliable for case-3 whereas CNN is needed in case-2. **(D)** Suspicion level caused by spatio-temporal continuity of SNN output. Suspicion level is used in determining the final fused position of prey.

Figure 4 shows the evolution of the algorithm through the cases outlined in the previous section. The inferences from both pipelines along with the final fused output can be seen in fusion demo-proof of concept^2^. The prey and predator start at a distance with the prey drone being out of the FoV of the predator (Figures 4A,B). This corresponds to the case-1. The SNN outputs in phase catch only the noise and stationary background and do not have spatio-temporal consistency. Therefore, the SNN outputs are incorrect in this part (Figure 4C). Convolutional neural network operates sparsely and CNN detections also verify that the prey is not present in the FoV. This causes the suspicion score to stay at zero (Figure 4D).

As the predator rotates, the prey appears within the Fov causing SNN to provide outputs that lie in the same region as the previous SNN outputs (case-2). This builds up the suspicion level for the SNN (Figure 4D–case 2). When the suspicion level exceeds the threshold, CNN is activated validating that the prey is present in the FoV. The suspicion level can be seen to go down quickly in this region for case-2. This is because the distance between the prey and predator is still high the SNN outputs are not very reliable.

As the distance between the predator and prey reduces, the system enters case-3 where rapid accurate outputs are required from the SNN with sparser CNN verification. This is reflected in the high suspicion level in this phase where spatio-temporally consistent outputs from the SNN cause the suspicion level to rise and stay high. Figure 3C also has correct SNN outputs in the region corresponding to case-3.

**Figure 5:**
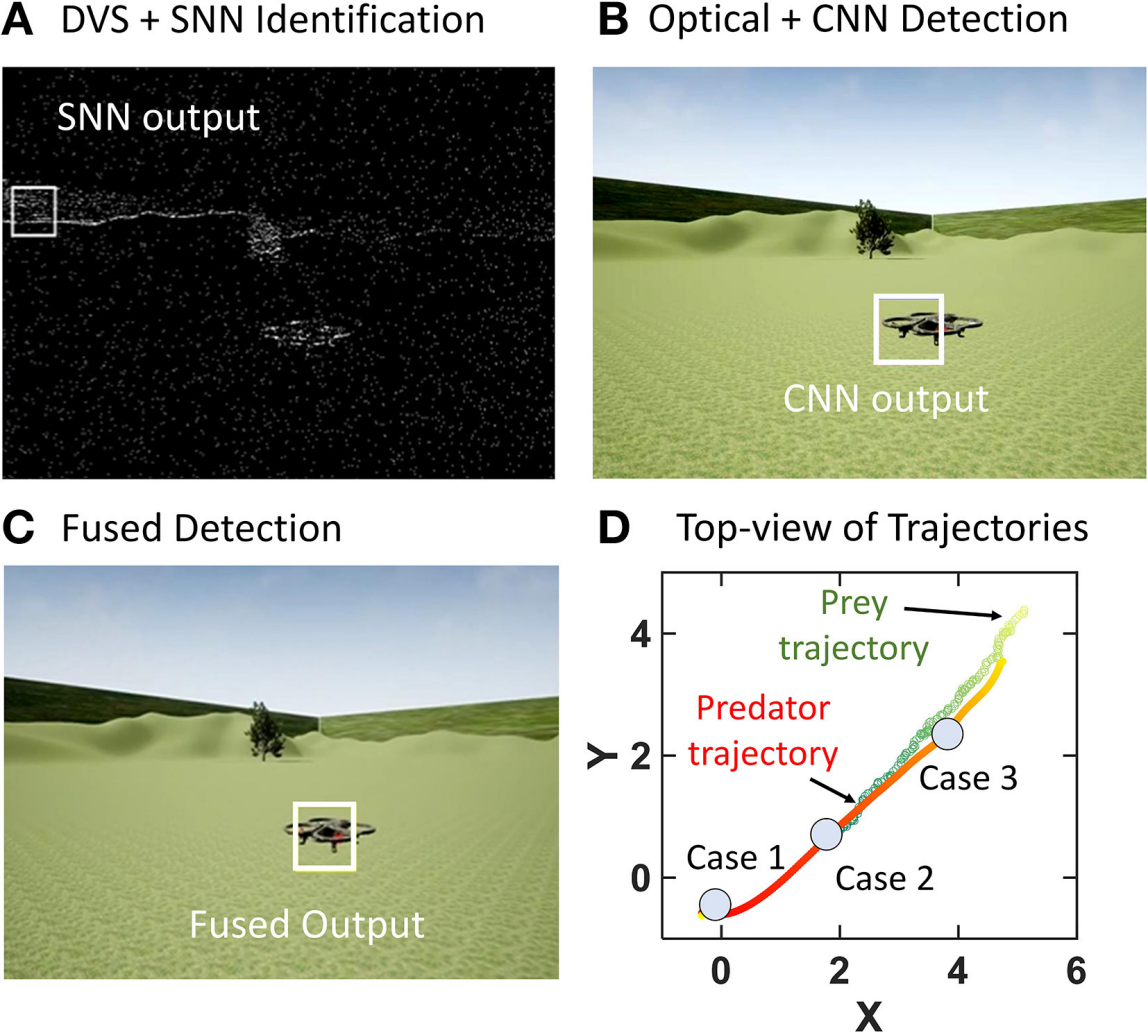
Illustration from an intermediate step in case 2 where an incorrect SNN output is ignored by the fusion algorithm to use the CNN output as the fused output. **(A)** SNN Output. **(B)** CNN output. **(C)** Target position after fusion using CNN output instead of noisy SNN output. **(D)** Top view of trajectory as predator goes through cases outlined in Section 2.3. The intensity of the colors corresponds to the time-step for both prey and predator.

Figure 5 illustrates an intermediate time step in case 2. The SNN detects inaccurate background objects (Figure 5A) while CNN has reliable detection (Figure 5B). The fusion algorithm corrects this as the final fused output uses CNN output (Figure 5C). Figure 5D shows the top view of the trajectories of prey and predator from the demo video denoting the regions of case 1–3 as the predator passes through them.

#### 3.1.2. Study in multiple environments and trajectories

The previous proof of concept is extended to two forest environments with sparse and dense backgrounds. The denser background is expected to create more self-motion caused events which in turn makes the SNN output noisier. The prey drone is programmed to fly with different evasive trajectories that make the prey enter the FoV for a brief period and escape. The high-speed fused (SNN+CNN) vision system is expected to be able to track these evasive trajectories. Both fused and CNN-only (frame pipeline only) systems are compared to establish the superiority of the fused system caused by the higher throughput provided by the SNN. The video demonstration for comparison is available at Multi-environment validation^3^. Interested readers are strongly encouraged to watch the video to understand the interplay between the frame and event pipelines.

**Figure 6:**
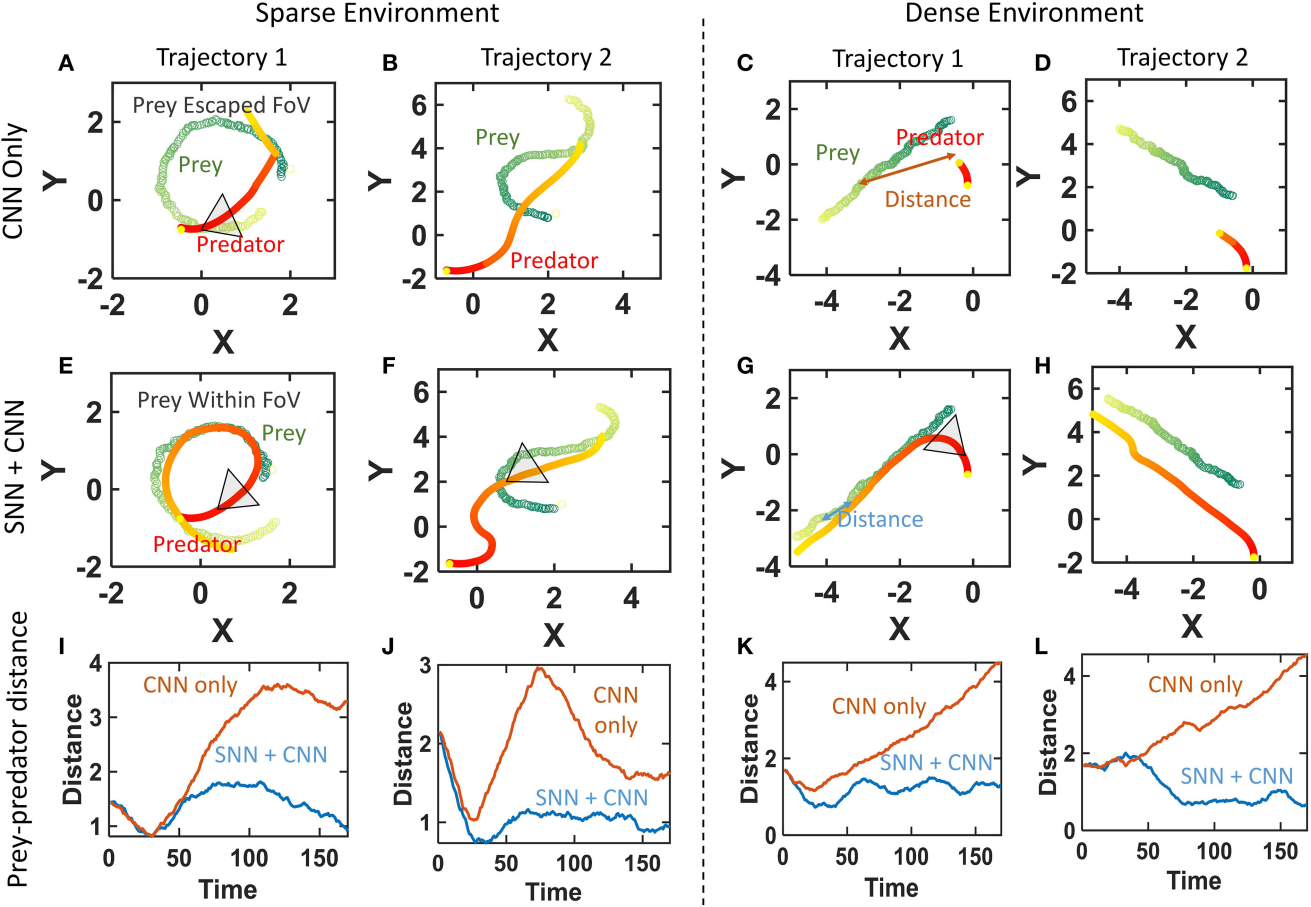
Performance improvement of the fused (SNN+CNN) system over CNN-only prey chasing for both sparse and dense environments. **(A–D)** Prey escapes the FoV as CNN throughput cannot keep up with the curvy prey trajectory. **(E–H)** Fused SNN+CNN tracks the prey using its higher speed while maintaining accuracy. **(I–L)** Distance between prey and predator diverges for CNN-only chasing while remaining low for the SNN+CNN system.

The representative final trajectories taken by the prey and predator in two of the trajectories in both environments are plotted in Figure 6. The prey can be seen to have a curvy trajectory as it tries to move out of the predator's view. The distance between the prey and predator as the algorithm progresses is plotted in the bottom sub-plots (Figures 6I–L). The CNN-only system is unable to keep up with these quick evasions and the prey moves out of the FoV for both sparse and dense environments (Figures 6A–D). This can be seen as the distance between the prey and predator rises for the CNN-only system at least once in the chase. The fused (SNN + CNN) system tracks the prey for a longer duration by keeping it within the FoV (Figures 6E–H). This maintains a small distance between the prey and predator as the predator chases the prey. We also notice a few runs of the fused system not being able to keep up and the prey escapes even with the higher frame rate. These experiments validate the potential of a fused system in having high-speed tracking while maintaining high accuracy.

We observe that the algorithm critically depends on the CNN detection for validating the SNN outputs. The failure cases typically correspond to the runs where the CNN does a mis-detection and they prey escapes. Thus, a reliable CNN is highly desirable. Secondly, the accuracy of SNN is low in the denser environment and causes the suspicion level to rise slower because of the mis-identifications. This sometimes causes the prey to escape. Incorrect CNN detection occurs more frequently in the cluttered denser environment. Therefore, the system is better suited for scenarios with smaller background clutter like outdoor high-altitude applications.

#### 3.1.3. Mitigating the accuracy vs. latency trade-off

**Figure 7:**
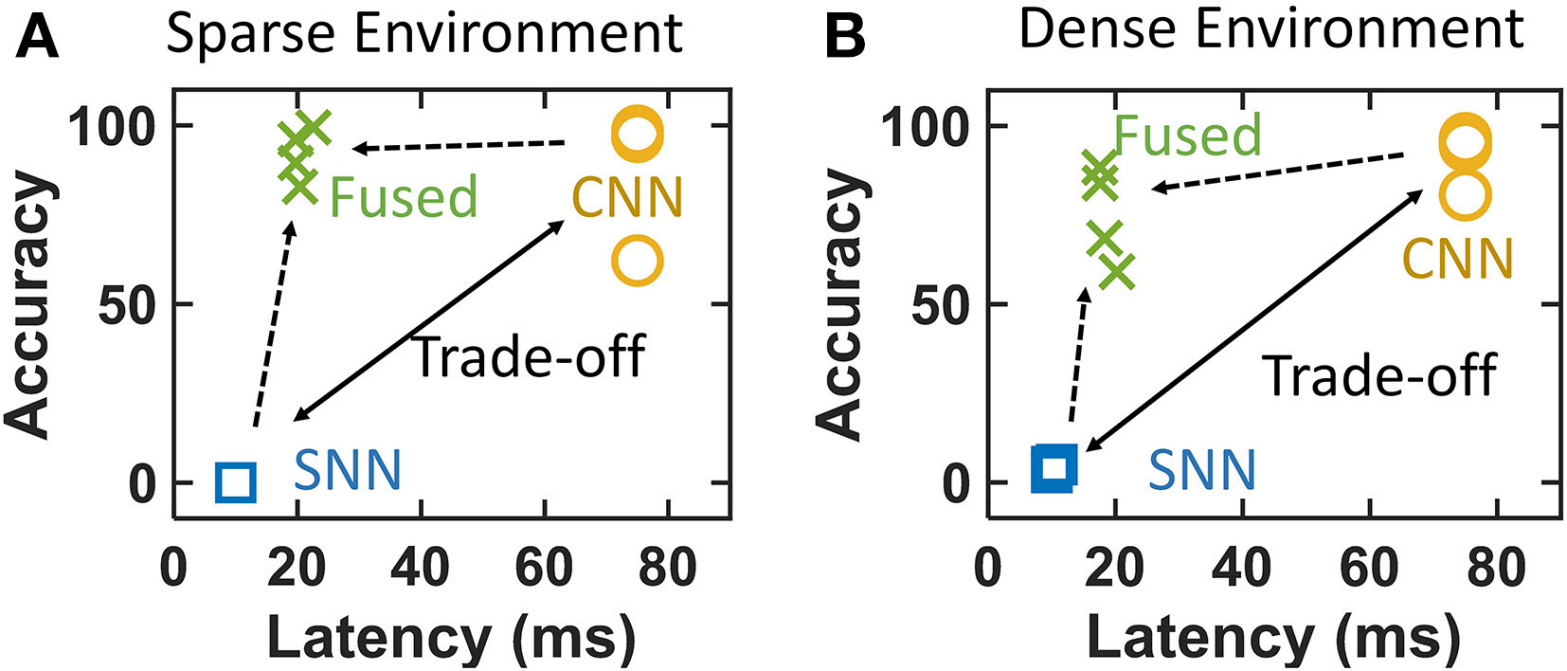
Mitigation of accuracy vs. latency trade-off in both **(A)** sparse and **(B)** dense environments. The dense environment provides lower relative fused accuracy compared to the sparse environment because of higher noise in SNN outputs.

We now assess the accuracy vs. latency trade-off in all 3 categories namely—SNN-only, CNN-only, and fused SNN+CNN. The SNN and fused detection provide a single point as output whereas the CNN provides a bounding box. The mid-point of the bounding box is taken as the CNN output. The accuracy for the SNN/CNN/fused results is calculated by checking if the predicted position is within a 50-pixel distance of the manually annotated position. Our accuracy metric checks if the predicted and actual position are within a similar region for actuating the predator drone to keep the prey within the FoV. Our closed-loop chasing uses the visual output at every time step to calculate the actuation velocities such that the prey gets centered within the FoV as the chasing progresses. This does not require exact bounding boxes and coarse localization (Lee et al., 2018; Zhang and Ma, 2021) provided by the single-point outputs is adequate. Other high-precision object detection approaches typically calculate the exact overlap between predicted and manually annotated bounding boxes in the image frame followed by evaluating mean average precision (mAP). However, we use center location error thresholding (50-pixels) instead of mAP as the comparison metric for the coarse single object localization task at hand. This center location error thresholding metric has also been used previously to calculate the accuracy of single object tracking (Wu et al., 2013) and chasing (Liu et al., 2016). We confirm the working of the system with such coarse detection system in the multi-environment demonstration video provided in the previous subsection. Figure 7 shows the accuracy and latencies obtained for four different trajectories shown in the video and three runs per trajectory for both virtual environments. Each point corresponds to the average accuracy for a trajectory. The latencies of SNN and CNN pipelines are extracted from hardware estimation described in Section 3.4.

Convolutional neural networks shows near-perfect accuracy with a longer latency (from section 3.3.2) as shown in Figure 7. Noisy outputs of the SNN-only system causes the prey to evade the predator in the initial time steps and it detects false positives once the prey exits the FoV. This causes SNN to have a very low accuracy. This causes the CNN and SNN pipelines occupy the positions of trade-off as shown in Figure 7 for both environments. The fused system compromises the accuracy slightly while maintaining small latency allowing efficient tracking even for quick evasive trajectories. The fusion algorithm reduces false alarms caused by noisy SNN while preserving the true positive outputs. The fused latency is calculated by dividing the total latency by the number of outputs from both SNN and CNN during entire execution of the operation. Thus, the accuracy vs. latency trade-off can be seen to be mitigated with a fused system with event + frame hybrid processing.

### 3.2. Real-world demonstrations

#### 3.2.1. Real-drones with emulated event data

The system was verified in both indoor and outdoor real-world settings as the next step. The DJI Tello Edu is used as a predator drone. This drone has a frame-based camera streaming the data to a local computer. The computer actuates the drone by processing the data through a wireless link. As the IMU readings are unavailable for these small drone, the actuation velocity of the previous step is used as the self-velocity in the current step for SNNs. Holystone 190S drone is used for prey which is flown manually. Conversion of frames to events takes a long time with the video interpolation strategy used in v2e. This makes the drones drift in the air with the wind and the inference takes a long time. To avoid this issue, we use the difference between the consecutive frames and threshold it to emulate the event accumulated frame. The communication of image and actuation velocities for the predator drone consumes 30 ms.

**Figure 8:**
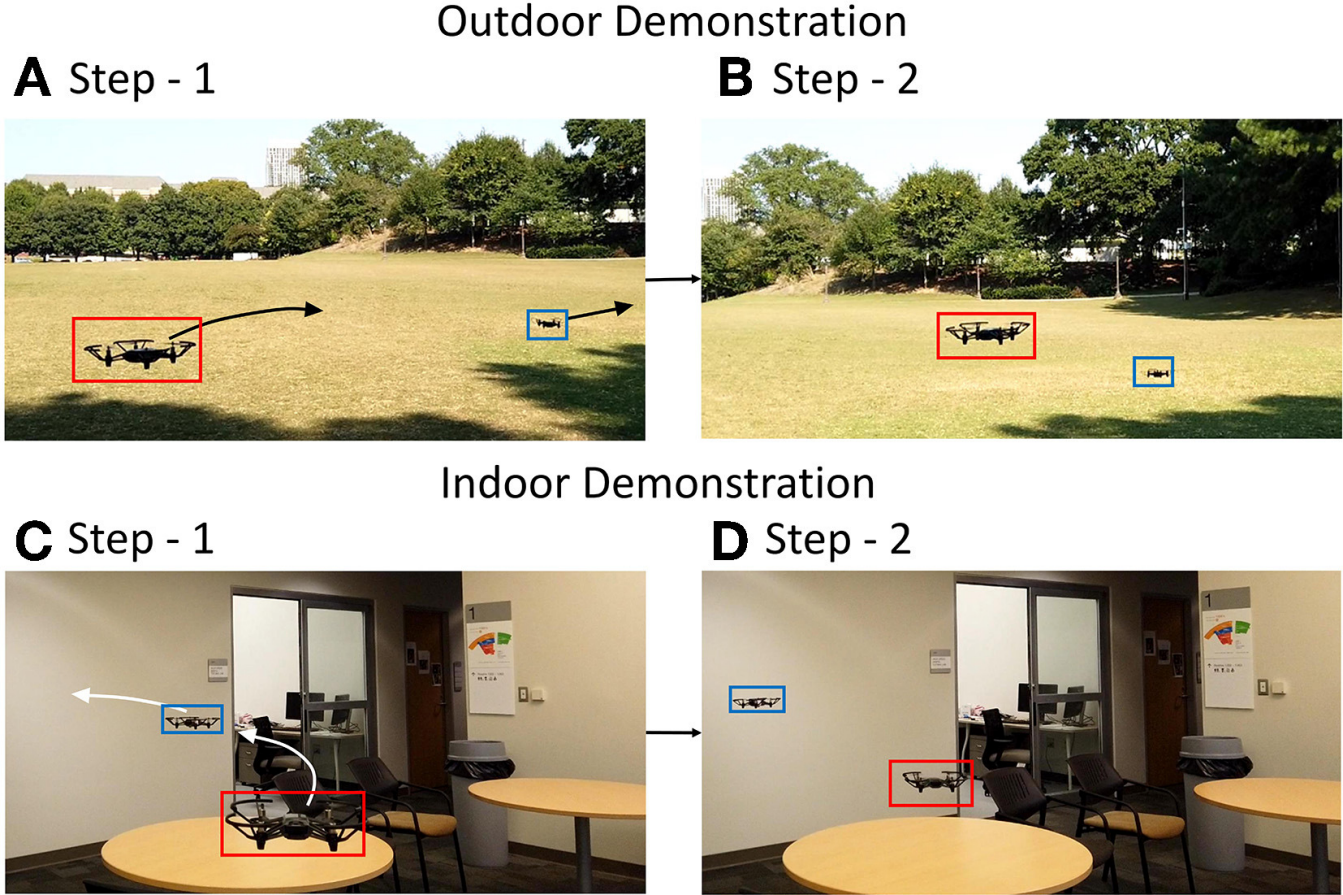
Screenshots from real-world experiments in **(A,B)** outdoor and **(C,D)** indoor scenario. The trajectories of the prey and predator are shown by the arrows with final positions in step-2.

Figure 8 shows the screenshots of the experiments recorded in videos— video-1^4^. The captured frames and detected drone positions can be seen in the video. Figures 8A,B shows the two steps in following the prey drone flying away while the predator drone autonomously follows it. Figures 8C,D shows the prey drone making a turn to evade the predator which eventually tracks it. This demonstrates the feasibility of the implementation of a closed-loop target tracking system. Although the realistic noise in DVS and IMU is not incorporated in these experiments, the multi-pipeline outputs are fused to generate an accurate inference. Desired chasing action from the predator drone demonstrates the potential of the system in a closed-loop setup.

#### 3.2.2. Hand-held DVS data

**Figure 9:**
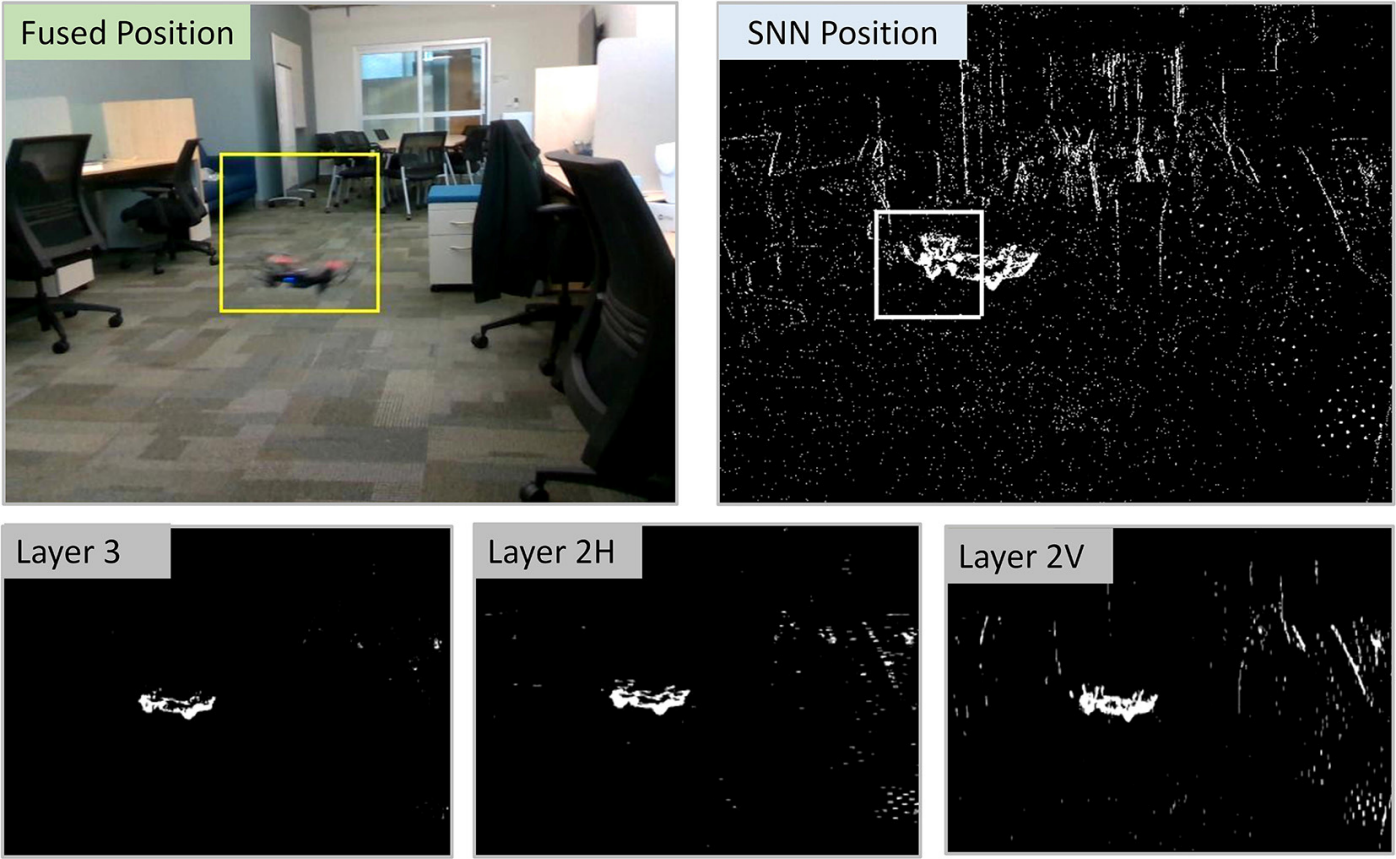
Screenshots from the processing of the data recorded using the multi-camera assembly. The spiking activity of the intermediate layers of the SNN can be seen to cause self-motion cancelation.

The experiments so far emulate the output of the DVS on a frame-like. However, real DVS data with real IMU provides significant noise that the system needs to tolerate. The depth and event camera do not align exactly and the robustness of the system needs to be tested for all these inherent inaccuracy of the real hardware. Therefore, we test the system on a real-data recorded on a hand-held DVS, depth camera, and the corresponding IMU readings. We use DVXplorer and Realsense d435i bound together as the camera assembly and the prey drone is flown manually in front of it in an indoor lab setting. Realsense camera provides IMU reading (62.5 Hz for accelerometer and 200 Hz for gyro-sensor). The self-velocities are calculated for rate-limiting 62.5 Hz and are used for SNN outputs until a new IMU reading is acquired The depth information is acquired at 90 FPS. Spiking neural network uses the previous depth information until a new depth frame is captured by the camera. This results in a slight lag between event and depth information if the operating throughput of SNN is higher than 90 FPS (264 FPS in this case). However, the SNN estimated position can be observed to be reliable with this lag as shown in video-2 The camera assembly is handheld and always points toward the prey drone. The drone escapes the FoV and re-enters. The captured data from DVS and the optical camera is aligned manually with simple linear translation and scaling of the image. The data is processed using the algorithm providing the outputs of CNN, SNN, and fused system. The details are available in this video—video-2^5^. A screenshot from the video is shown in Figure 9. The spiking activity of the layers of SNN shows how ego-motion cancelation results in the activity corresponding to the prey to survive. The algorithm can be seen to work even in the highly cluttered indoor setting with reasonable accuracy. The system uses the faster SNN outputs along with the CNN outputs to boost the throughput of the overall system. Even though this system does not close the loop with autonomous actuation, the working of the system with real data predicts that it is capable of running on an aerial platform. The accuracy can be improved further by building event + frame datasets for object tracking using mobile platforms. Training SNN using such datasets may improve the overall accuracy of the system. A future step would involve mounting the assembly on a drone to close the loop from sensing to actuation.

### 3.3. Design space exploration

The design parameters like “span,” noise in self-velocity affect the SNN output. In addition to this, the selection of epoch duration determines the SNN latency and throughput and presents an internal accuracy vs. latency trade-off for the SNNs. For very short epoch intervals (for high throughput), inadequate number of events are processed injecting noise. This causes lower accuracy. For lower throughput for SNN, higher accuracy is achievable. On the other hand, the feature detection model and object detection method in the CNN pipeline presents another accuracy vs. latency trade-off within the CNN pipeline. Large CNN models typically have larger accuracy at the cost of slower execution. All these parameters and design variables offer a wide range of parameters to choose from. We explore these design choices in this section. The optimal parameters observed in this section are used in the experiments presented in the previous discussion.

#### 3.3.1. Parameter tuning for event pipeline

“Span” and the noise in self-velocity directly affect the spiking pattern in the SNN. The exact self-velocity of the predator is available in the simulation environment whereas it is noisy when acquired as the accumulated accelerometer sensors output in the real IMU data. Therefore, we use simulations in the virtual environment for finding the optimal values for these parameters and their effect on the accuracy of SNN output. We also investigate if the fused SNN + CNN system is capable of improving the accuracy for these empirical parameters. The experiments are carried out for the trajectory shown in Figure 4D.

**Figure 10:**
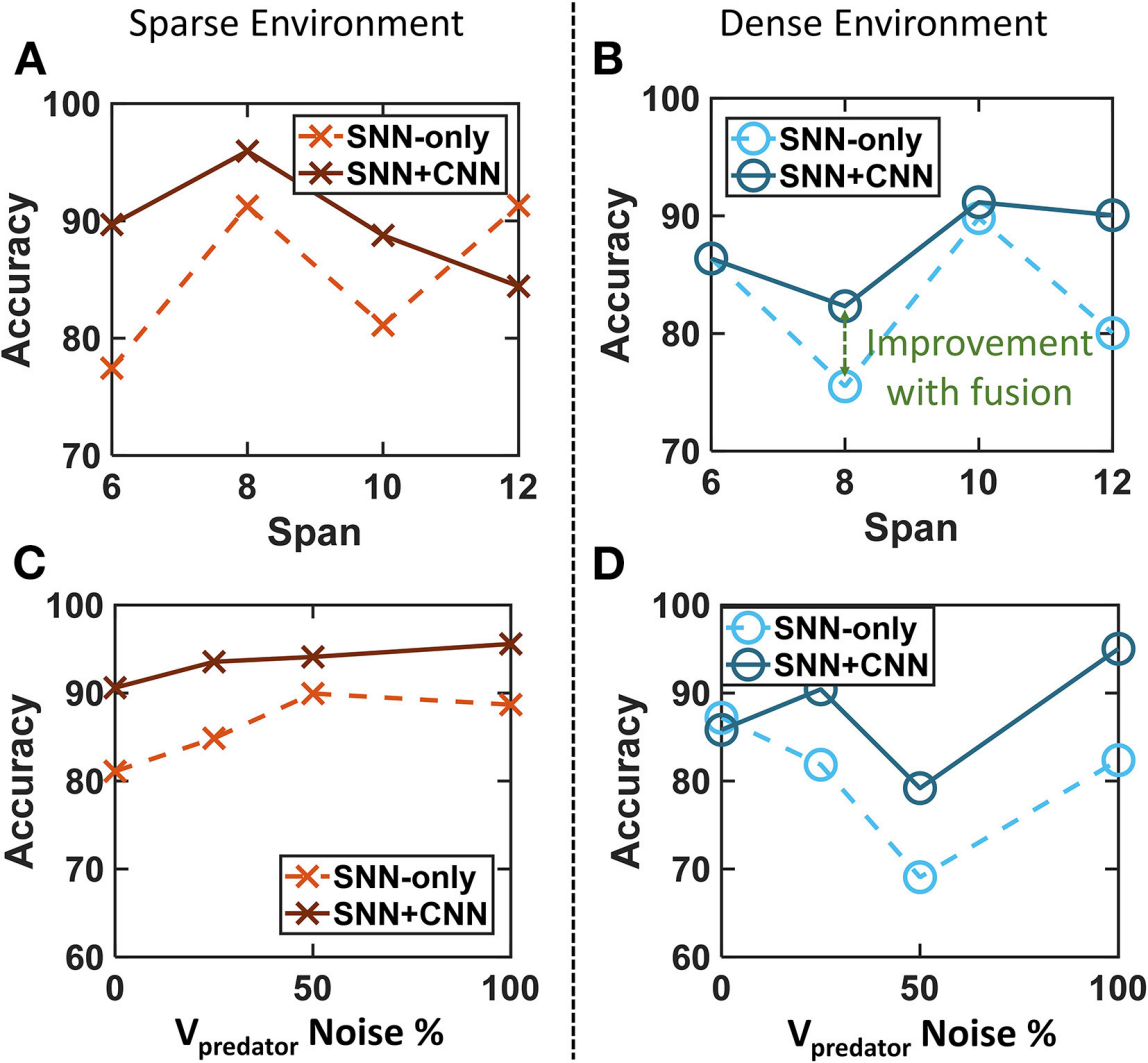
Tuning the empirical parameters for SNN filter and fusion algorithm. **(A,B)** Target localization accuracy with a varying span of connectivity for both sparse and dense environments. The span of 10 is used for higher accuracy. **(C,D)** Target localization accuracy while varying the induced noise in the predator's velocity for both sparse and dense environments. The final fused accuracy is robust to noise in self-velocity.

- Span: In the first experiment, the span of connectivity between layer 1 and layer 2 is swept from 6 to 12 in the steps of 2. A higher span indicates higher injected activation in layer 2 for every incoming event from the DVS. This results in a high chance of spiking in layer 2 and thus a higher probability of finding persistent activity. However, the chance of mistaking a steady object for the target also increases with higher activity injection. Thus, both false positive and true positive outputs rise as the span is increased. Three experiments are carried out for each combination and both sparse and dense trajectories. The results are plotted in Figures 10A,B. The accuracy can be seen to improve from SNN-only identifications to SNN+CNN fusion for most of the data points. We use the span of 10 as it provides higher relative accuracy in both sparse and dense environments.
- Noise in Self-velocity: Accurate reading of self-velocity plays a key role in the self-motion cancelation network. This bio-inspired approach relies on the assumption that the IMU sensors can provide an accurate estimate of the pose and speed. However, the sensors are often noisy in a real-world scenario and it is necessary to test the limits on error tolerance. We add noise in the velocity

(3) $$\begin{array}{l} V_{noisy}=V_{actual}\left(1\pm noise/100\right) \end{array}$$

The noisy simulations affect the accuracy of SNN. Figures 10C,D shows that a high percentage of velocity noise can be tolerated by the algorithm highlighting its robustness. The SNN-only accuracy is lower compared to fused accuracy with CNN validations boosting the accuracy. The degradation in accuracy for SNNs is more for the dense cluttered environment as expected.

The simulations show that both span and the noise in self-velocity have a weak correlation with the accuracy of the event pipeline. However, the accuracy improves significantly after fusion with CNN output as noisy SNN estimates are eliminated. Additional exploration using real DVS data with accurate pose estimation in different environments can be carried out in the future.

**Figure 11:**
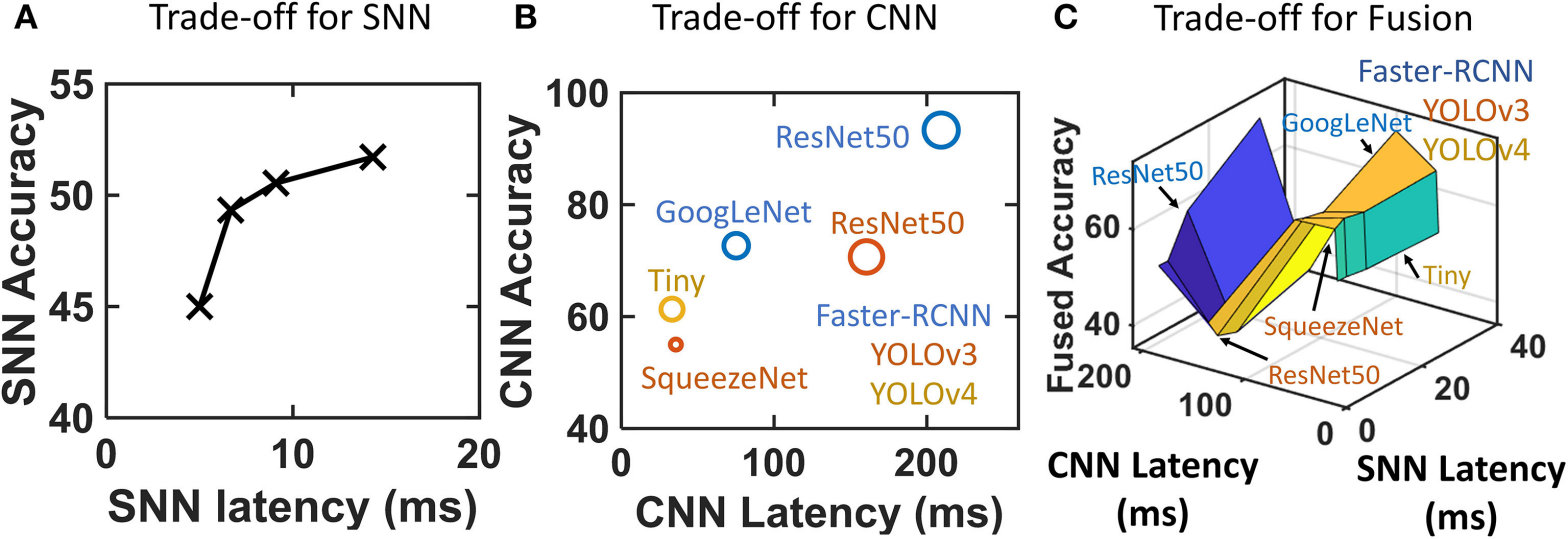
Both event and frame pipelines have internal accuracy vs. latency trade-offs. **(A)** Accuracy of the event pipeline increases when the epoch duration is large (lower throughput) with more events to infer from. **(B)** Different feature extractors and object detectors cause performance trade-offs for CNN. The color coding shows the detector while the feature extractor is denoted in the figure. Resnet50+FasterRCNN is the most accurate while Squeezenet+YOLO is the fastest. **(C)** Fused accuracy requires an accurate CNN with reasonably high speed for high accuracy. The latency of SNN has a relatively low impact on fused accuracy while it determines the throughput. GoogleNet+FasterRCNN is the most suitable.

- Epoch Duration (SNN Latency): The epoch duration in SNN controls the accuracy and latency of the event pipeline. The events generated within an epoch duration are used to generate an SNN inference. Therefore, the epoch duration controls the SNN throughput and latency. This experiment cannot be reliably carried out in the virtual environment, because v2e (Hu et al., 2021) reports a simulated time stamps. Therefore, the experiment is carried out using the real DVS data from Section 3.2.2. The data is manually labeled for the position of the prey. The latency of an epoch is varied in Figure 11A to find the accuracy of SNN (event pipeline). A smaller duration of epoch results in a higher throughput for SNN. The plot shows that the accuracy monotonically increases for a larger epoch duration. This indicates that smaller epoch duration causes a small number of events to generate an inference from. This results in more noise injection and a reduction in the accuracy. A longer epoch produces large number of events required for a reliable output. High SNN throughput results in more SNN outputs between every consecutive CNN detection. An effect of this on the final fused accuracy is explored next.

The virtual environments used in this case alter the amount of background clutter and show similar trends in the hyperparameters. Therefore, we expect the trends to hold for other scenarios with similar testing setups. However, if the setup changes drastically, e.g., very high-speed chasing in a high-altitude environment the tuning may need to be carried out again.

#### 3.3.2. Model selection for frame pipeline

The CNN needs to detect the prey drone accurately and quickly for accurate fusion. In case of an incorrect detection, the SNN identifications after it rely on it for updating the suspicion level and the subsequent outputs result in accuracy degradation. Therefore, a high accuracy is desirable. Simultaneously, if the CNN is too slow, then multiple SNN outputs get processed within two consecutive CNN outputs inducing inaccuracy in the final fused output. The key requirement for CNN here is the ability to track small drones. This is because the setup is completely dependent upon the CNN when the prey is far away corresponding to case-2. Thus, a reliable, fast, and small object detection capable CNN is required. A previous survey on small object detection dataset (Chen et al., 2016; Pham et al., 2017; Nguyen et al., 2020) shows YOLO and Faster-RCNN have higher accuracy compared to single-shot detectors. The size of the feature detection backbone also plays a key role in the accuracy and latency of CNN. Thus, the design space consists of multiple object detection methods and feature extraction networks to choose from.

First, we train multiple models and find their respective accuracy. We use the data recorded from the hand-held camera assembly that records both event-stream and frames for the flying prey drone simultaneously. The image frames from this dataset are manually labeled. The data consists of 1,200 training images and is validated on a video consisting of 400 frames. Additionally, images from Lin (2020) and Gupta (2020) are added for a diverse training. The pre-trained feature extraction networks trained on the Imagenet dataset are used from Matlab. The networks are trained and tested to find the accuracy shown in Figure 11B. The accuracy for large feature extraction networks like ResNet50 is higher than the smaller networks as expected. Faster-RCNN detectors have higher accuracy as observed in previous literature (Nguyen et al., 2020; Pham et al., 2017). This is because of the small size of the target prey drone and faster-RCNNs are better suited for small object detection.

In the second step, we calculate the latency of each of the networks on an edge-FPGA of Zync-7000 (explained in Section 3.4). We use ScaleSim (Samajdar et al., 2018) as the architectural simulator for latency characterization. ScaleSim has a systolic CNN array architecture. We characterize it as per Zynq-7000 SoC's resource availability. ScaleSim supports resources as powers of two seamlessly. Therefore, 400 DSPs are planned to be used in 16 × 16 systolic configuration. Similarly, 265 kB BRAM (local memory) is mapped onto 256 kB SRAM cache. The input size and layer sizes for the network are provided as input and the execution latency for a single image is extracted as the output of the network. The latency is plotted across the accuracy values as shown in Figure 11B. Squeezenet for YOLOv3 being small networks have a low inference latency whereas the ResNet50 on Faster-RCNN takes a longer time to infer. This plot also reveals the accuracy vs. latency trade-off within CNNs that motivates this work. It can be seen that even the fastest CNN is unable to provide very high throughput (>100 FPS) showing the need for the event pipeline.

#### 3.3.3. Parameter selection for fusion algorithm

The accuracy vs. latency trade-off within both SNN and CNN pipelines affects the performance at the fused outputs (Figure 11C). We run the fusion algorithm on the camera assembly data from Section 3.2.2. The overall accuracy of the fused system is plotted across individual SNN and CNN latencies. The final accuracy after fusion can be seen to be critically dependent upon the CNN model. GoogleNet+FasterRCNN provides the highest final accuracy. This is because this configuration achieves the optimal balance between accuracy and latency. ResNet50+FasterRCNN has very high accuracy but longer latency causes incorrect SNN outputs to leak in between consecutive CNN inferences. This degrades the overall fused latency for the ResNet50+FasterRCNN setup. ResNet50+YOLO has worse fused accuracy compared to squeezenet because of its longer inference latency in spite of being slightly more accurate. This study shows that both accuracy and latency on the CNN model are of key importance in the final fused accuracy.

Spiking neural network latency determines the overall throughput of the network and also controls the accuracy of the SNN pipeline as seen in Figure 11A. However, it does not have a critical impact on the overall fused accuracy of the system. This shows that CNN model selection is imperative in determining the fused accuracy of the system whereas SNN latency is important in the final throughput of the system. The previous results use the parameters tuned in this section. This study provides a methodology to evaluate the choice of the best model and SNN parameters corresponding to a processing platform. Our Zynq-7000 FPGA analysis focuses on edge-compute. A larger FPGA can reduce the inference latencies for all CNN architectures and therefore the choice of the best network may differ. An exhaustive analysis of multiple compute platforms, object detection architectures, and backbone networks may be taken up in the future.

### 3.4. Throughput estimation

The system requires a low-power (< 10 W) edge application at a high speed. It requires support for a highly compute-intensive CNN with multi-channel convolution, as well as memory-intensive SNN requiring membrane potential storage and update for a large number of neurons. Thus, the hardware requires parallelization for faster CNN and block-wise memory availability for SNN. The edge-TPU suits well for CNN but does not support the high-speed requirement of the SNN. Similarly, a dedicated SNN accelerator like Loihi (Davies et al., 2018) cannot map the CNN effectively. Using individual optimized boards requires additional effort in synchronization of the data and adds latency of communication between the boards. Thus, a programmable FPGA offers the optimal trade-off point in the hardware space with decent support to both pipelines as well as low-power edge applications. Spartan FPGA family lies in the required low power range but has very limited resources. Thus, we use Zynq 7000 FPGA for hardware mapping (BERTEN, 2016).

**Figure 12:**
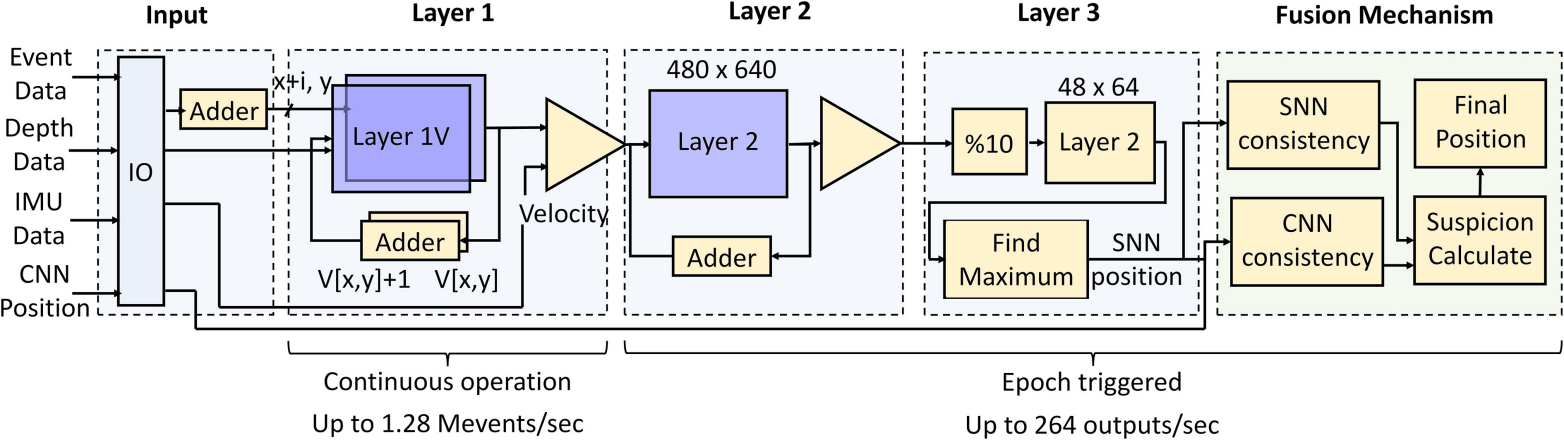
FPGA micro-architecture for throughput controlling event pipeline and fusion algorithm. The execution of layers 2, 3 and the fusion algorithm determines the maximum potential throughput of 264 outputs per second. The asynchronous layer-1 has the capacity of handling 1.28 × 10^6^ events per second.

The SNN and fusion pipeline controls the maximum throughput of the network. The micro-architecture of the SNN and fusion system is shown in Figure 12. The input from the event camera, IMU, and depth camera is acquired at the input layer from the IO. The output of the CNN pipeline is assume to be acquired from an internal CNN block running the CNN. Layer 1 requires asynchronous operation as outlined in Section 2.1 while the next layers along with the fusion algorithm operate after every time epoch. Both layer 1H and 1V are to be implemented in a block RAM for quick access to incoming event packets. This makes the SNN design memory intensive for storing 480 × 640 (frame size) activations. The IF neurons add up the event activity and store the spiking information for the next layers to process it. A counter triggers layers 2 and 3 after the duration of an epoch to identify the position. Thus, the minimum epoch duration (maximum SNN throughput) depends upon the latency of execution of layers 2, 3, and fusion algorithm together.

We implement the above architecture using Vitis High-level Synthesis on Zynq 7000 SoC (xc7z035-fbg767-1). All SNN layers along with the fusion algorithm are mapped onto the FPGA. The FPGA is operated at a clock period of 12 ns which is the maximum allowed clock frequency provided by the synthesis. Layer-1 takes 65 clock cycles per incoming event including the spike generation. Thus, 780 ns are taken for every incoming event allowing the processing of 1.28 M event/s. Execution of layers 2, 3, and fusion algorithm takes 3.78 ms. Therefore, the minimum epoch duration is 3.78 ms with a maximum throughput of 264 FPS. This confirms that a straightforward implementation on an edge-FPGA is able to provide humongous throughput for the SNN. The resources consumed by the implementation above are 375 BRAM (75%), 1 digital signal processor (DSP) (0.1%), 1,073 flip flops (FF) (0.3%), 1,782 Look-up Tables (LUT) (1%) showing low resource consumption on board. The SNN implementation is memory intensive whereas the CNN implementation is generally DSP intensive with multiple parallel operations. Thus, we expect complementary resource consumption by the event and frame pipelines directly suitable for FPGAs. An end-to-end bandwidth optimized implementation of both pipelines can be taken up in near future.

Drone navigation typically uses companion computers for vision processing that communicate the commands for actuation to the flight controller that in turn drives the motors. Autopilot software-hardware stacks like PX4 use UART communication for receiving the actuation commands. The maximum rate of communication lies in the kHz range. Therefore, our throughput of 264 outputs per second is not redundant from the electronics perspective and further improvement is also desirable. From the mechanical perspective, customized mid-sized drones capable of carrying the weight of the DVS, frame camera, and compute platform are shown in Zhu et al. (2018) and Falanga et al. (2020). These drones are demonstrated to move at ~ 2 m/s. This corresponds to an SNN output for every sub-centimeter displacement which would be sufficient for tracking problems. High-speed drones are typically lightweight and are unable to support large weights of the cameras and compute assembly. A closed-loop study of altering the sensor and compute weight on customized drones could enable the search for the optimal point for the maximum speed of the drone vs. sensor and compute weight. This can be taken up in the future.

### 3.5. Comparison with prior work

We compare our method with previous demonstrations of high-speed target localization (Table 1). YOLOv3 works with a frame camera and performs reasonably fast (Redmon and Farhadi, 2018) but works on a power-intensive GPU. Vibe (Van Droogenbroeck and Barnich, 2014) works with the frame difference between consecutive frames to identify the motion but is eventually limited by the frame rate of the camera. The approaches using event cameras typically show non-selective identifications and tracking. This means that all moving objects are identified without being selective. Falanga et al. (2020) uses optical flow and event time stamp information to segregate the moving object. Other non-selective tracking approaches (Mitrokhin et al., 2018; Zhou et al., 2021; Vasco et al., 2017) use an energy minimizing optimization to find the 3D movement of event clusters and find outliers in them to be classified as moving objects. These non-selective methods are incomplete without an added object distinguishing network. Additionally, the latency of these optimizations is speculated to be typically higher (Mitrokhin et al., 2018) compared to our SNN because of more complex iterations. Convolutional neural networks have also been used with modified objective functions for segmentation of the scene into multiple objects (Stoffregen et al., 2019; Alonso and Murillo, 2019). But the setup becomes computationally expensive because of the convolutional backbone and the speed may be compromised on an edge platform. A fused optical and event-based localization capability is used in Yang (2019) but requires a Tianjic neuromorphic ASIC. Our method shows a high throughput using SNNs and accurate and selective detection of prey drones using CNN. Thus, our method can provide a high-speed implementation on an edge-platform suited for UAV applications.

**Table 1:** Previous work on high-speed target localization.

**Reference**	**Camera**	**Platform**	**Time**	**Target**
			**ms**	**Selective**
YOLOv3, Redmon and Farhadi (2018)	Optical	Titan X GPU	45	Yes
Vibe, Van Droogenbroeck and Barnich (2014)	Optical	CPU T7300	599	No
Falanga et al. (2020)	DVS	Jetson TX2	3.5	No
Mitrokhin et al. (2018)	DVS	Intel i7 CPU	10	No
This work	Both	Zynq FPGA	3.78	Yes

## 4. Discussion

### 4.1. Bio-inspired ego-motion cancelation

A key contribution of this work lies in the design of the ego-motion filter using SNN inspired by neuro-biological advances in recent years. The nullification of self-generated action (reafference) finds ample examples in biology. Male crickets cancel their chirp preventing them to respond to it (Kim et al., 2015). Electric fish cancel the electric field generated by their own actions (Kim et al., 2015). In primates, inputs from the vestibular system are processed in the cerebellum to keep track of the motion (Cullen et al., 2011). Recent progress in neuroscience postulated the presence of differentially weighed neural connections behind this phenomenon (Zhang and Bodznick, 2008). The first neurophysiological evidence for this is found as a distinct class of neurons in the vestibular nucleus of the primate brainstem (Oman and Cullen, 2014). Another model argued that when the estimated response of an ego-action is close to the perceived action, the cancelation happens through adaptive inhibitory circuitry (Benazet et al., 2016). A similar observation was made earlier for humans where “smooth pursuit eye movement” for a target moving in a direction decreases the sensitivity of the vision for the opposite direction (Lindner et al., 2001). The behavioral experiments argue that locomotive insects send a copy of their reafference perceived by the sense to an internal neuron circuitry for cancelation. The key experimental study in the ego-motion cancelation in the vision on drosophila (housefly) is recently published where the neurons corresponding to optical flow around yaw and pitch axis are probed (Kim et al., 2015). This shows that the visual neurons received the motor-related inputs in-flight turns causing the visual inputs to be strongly suppressed. This is very similar to the method we propose where we have the visual response cancelation using the vestibular ego-motion using inhibitory synapses (differential cancelation). We showed this neuro-inspired network is capable of detecting the prey with high confidence when it is close to the predator for high-speed response.

### 4.2. Neuro-mimetic multi-pathway processing

Our system is inspired by the multi-pathway model of the visual processing proposed and found in many animals. Multiple neural paths specialize in specific tasks and combine their inferences. The wavelength insensitive neurons are observed to work for regular vision but UV sensitive neurons work for prey tracking and foreground cancelation for larvae zebrafish (Zhou et al., 2020). It has been stated that the color-intensive pathway in the brain is slower compared to grayscale but richer in spatial details of the information (Gegenfurtner and Hawken, 1996). Monkeys have visual pathways optimized for global slow and locally fast signals for high-speed tracking (Mazade et al., 2019) (similar to our work). Houseflies also process local and global motion data separately (Gollisch and Meister, 2010). Humans have rods and cones in the retina separating color vision from grayscale activity at the beginning of the processing pipeline. The motion and color-sensitive pathways were suggested to be different in housefly (Yamaguchi et al., 2008). This matches with our design where spatially detailed color information (frame pipeline) and temporally fine event information (event pipeline) are gathered separately and processed in separate pathways before merging into the fusion algorithm. Another feature of our work is that SNN and CNN are suited for different phases of chasing (cases 1–3). This has a parallel where different neuronal clusters are observed to be active in different stages of hunting for zebrafish (Förster et al., 2020). When the predator is at a distance and following the prey, a set of neurons suited for small object detection and tracking are active. However, as the prey is approached and becomes bigger in size different sets of neurons take over the detection task. Therefore, merging and cooperation between the neural paths may have even more interesting insights and applications in the future.

### 4.3. Usage of hard-coded networks

Our SNN takes a rigid synaptic weight structure processing the asynchronous incoming event stream for canceling the ego-motion. A natural criticism about it can be a lack of training methodology to allow learning. However, many instinctive tasks have been observed in insects which are postulated to be shaped by evolution without a learning response (Kanzaki, 1996). Furthermore, the plasticity is high in the initial phase of life and then converges to learnt behaviors after the neural development is near completion (Arcos-Burgos et al., 2019). The argument that most of the animal behavior is encoded in the genome instead of being learned (Zador, 2019) also supports this approach. Hard-coded SNNs have been used with with event-cameras for numerous tasks like stereo depth estimation (Osswald et al., 2017), optical flow computation (Orchard et al., 2013), lane-keeping (Bing et al., 2018), and looming object avoidance (Salt et al., 2017). We believe that the accuracy of our network can be improved with SNNs trained for drone detection. This provides the first-order demonstration of shallow and fast computation of ego-motion cancelation as a step in building bio-inspired SNN robots for high-speed applications.

### 4.4. Other related works

Simultaneous use of event and optical camera has been approached in Liu et al. (2016) for predation task in wheeled robots as well. This simultaneous event and frame-based approach uses an event camera to identify the region of interest while CNN does the object recognition on the identified region saving energy consumption and boosting the processing speed. However, the CNN latency for a single frame processing persists. The region of interest identification task becomes challenging with the cluttered background that we utilize in our work, limiting the performance of this system. Another hybrid approach has been used in a fused SNN + CNN approach for optical flow calculation (Lee et al., 2021). The events are accumulated using SNN and are merged into a CNN for more accurate optical flow calculation. However, the CNN backbone remains critical for every inference and the throughput gets eventually limited by the compute. Our approach has the independent frame and event-based pipelines similar to Lele and Raychowdhury (2022) that only provide their respective outputs for the fusion algorithm which works in linear time.

Event camera-based moving object tracking problem has also been addressed using model-based approaches like cluster detection (Delbruck and Lang, 2013), corner detection (Vasco et al., 2016), ICP (Ni et al., 2012), region of interest tracking (Mohan et al., 2022), etc. However, these works operate with either a stationary camera or stationary environment as opposed to independently moving prey and predator in this case. A modification to the region proposal algorithm to identify the independently moving object from velocity estimation can be incorporated to allow tracking using a moving predator platform. Combining these approaches with hybrid processing may open up interesting future directions.

### 4.5. Potential limitations

It is worthwhile to speculate on the limitations of the proposed system. The performance assumes both pipelines to be working reliably for interdependent cooperation. Therefore, reasonable lighting conditions would be required for the CNN pipeline although event cameras are known to work in low-light environments. The stability of the drone under windy conditions where the drone drifts creating spurious activity will require accurate IMU sensors for ego-motion cancelation. Vibrations of drone frames can also corrupt the event stream and IMU data. Therefore, a stable flight is desirable for the accurate functioning of the SNN filter. High altitude flight is expected to be easier with sparser occlusions. We observe that the rapid motion of prey drones causes image blur in the frame-based camera corrupting the CNN output. Therefore, a high-quality image acquisition or image stabilization mechanism may be needed in ultra-rapid response implementations. Histogram-based method utilized in SNN filter may get limited if directly applied to simultaneous tracking of multiple objects. Recent works have demonstrated region proposal on low-cost event-accumulated binary images followed by multi-object tracking even in presence of occlusion showing low computation and memory costs (Acharya et al., 2019; Mohan et al., 2022). Customized circuits for this application (Bose and Basu, 2022) demonstrate high throughput and energy efficiency. Such methods can be applied for multi-object tracking in place of layer-4 after canceling the activity caused by the self-motion. Finally, selective tracking of an object from multiple moving targets can be addressed in the future by altering the spatio-temporal filtering algorithm to handle the position from multiple SNN and CNN outputs.

### 4.6. Hardware implementation

Numerous interesting possibilities for circuit implementation for such hybrid systems are also possible. We evaluated a hybrid processing method with FPGA. However, the latency of memory access and clocked sequential nature of FPGA limits the performance of SNN. Dedicated asynchronous SNN hardware like Loihi, truenorth (Akopyan et al., 2015; Davies et al., 2018) would overcome the bottleneck allowing massive parallelism with very low power. However, these general-purpose SNN ASICs have a large hardware overhead for the relatively simple network that we propose. Processing the entire flow of the algorithm on a single die with optimized circuits will allow the exploitation of a truly hybrid framework from sensing to implementation at the constrained power budget. Non-volatile crossbar arrays like resistive RAM also show high throughput and low-power CNN processing capability (Chang et al., 2022) that can be augmented with on-chip SNNs. Additional exploration in this direction needs to be taken up in the future.

## 5. Conclusion

We proposed a visual target localization system that leverages the fusion of frame and event-based cameras with corresponding processing neural networks to attain the accuracy and latency advantages simultaneously. The ego-motion canceling SNN and object detecting CNN exploit the temporal and spatial resolution of the respective sensors in two independent pipelines. The SNN filter incorporates the connectivity from the insect brains and multi-pipeline processing and interplay between SNN and CNN has a neuro-biological basis in primate and insect brains. The system is shown to work using a virtual environment and real-world demonstrations. The feasibility of implementation on a low-resource FPGA shows a potential throughput of 264 FPS. This work may open exciting possibilities in building hybrid SNN systems to mitigate the fundamental issues in frame-based processing.

## Data availability statement

The raw data supporting the conclusions of this article will be made available by the authors, without undue reservation.

## Author contributions

AL wrote the manuscript and performed the simulations and experiments. YF helped with the experiments and concept development. AA designed the simulation environments and assisted in simulations. AR helped with developing the concept, refining the experiments, and writing the manuscript. All authors contributed to the article and approved the submitted version.

## Funding

This work was supported by CBRIC, one of six centers in JUMP, a Semiconductor Research Corporation (SRC) program sponsored by DARPA, and NSF grant CCF-2153440.

## Conflict of interest

The authors declare that the research was conducted in the absence of any commercial or financial relationships that could be construed as a potential conflict of interest.

## Publisher's note

All claims expressed in this article are solely those of the authors and do not necessarily represent those of their affiliated organizations, or those of the publisher, the editors and the reviewers. Any product that may be evaluated in this article, or claim that may be made by its manufacturer, is not guaranteed or endorsed by the publisher.

## Footnotes

^2^ https://youtu.be/wO86TO5PL6U

^3^ https://youtu.be/cQ9OGRgmv3w

^4^ https://youtu.be/IJgoaO6n-NY

^5^ https://youtu.be/aZsX4heR2gw
